# Supplementary material for: HIV self-test performance among female sex workers in Kampala, Uganda: a cross-sectional study
Source: BMJ Open. 2018 Nov 8;8(11):e022652. doi: 10.1136/bmjopen-2018-022652 (PMC6231563; doi:10.1136/bmjopen-2018-022652)
Supplement: Author's manuscript [file bmjopen-2018-022652.draft_revisions.pdf]

BMJ Open is committed to open peer review. As part of this commitment we make the peer review history of every article we publish publicly available.

When an article is published we post the peer reviewers' comments and the authors' responses online. We also post the versions of the paper that were used during peer review. These are the versions that the peer review comments apply to.

The versions of the paper that follow are the versions that were submitted during the peer review process. They are not the versions of record or the final published versions. They should not be cited or distributed as the published version of this manuscript.

BMJ Open is an open access journal and the full, final, typeset and author-corrected version of record of the manuscript is available on our site with no access controls, subscription charges or pay-per-view fees (http://bmjopen.bmj.com).

If you have any questions on BMJ Open's open peer review process please email editorial.bmjopen@bmj.com

BMJ Open

HIV self-testing process accuracy and results interpretation among female sex workers in Kampala, Uganda: a field assessment

Journal:	*BMJ Open*
Manuscript ID	bmjopen-2018-022652
Article Type:	Research
Date Submitted by the Author:	02-Mar-2018

Complete List of Authors:	Ortblad, Katrina; Harvard T.H. Chan School of Public Health, Global Health and Population<br>Kibuuka Musoke , Daniel ; International Research Consortium<br>Ngabirano , Thomson ; Uganda Health Marketing Group<br>Nakitende, Aidah ; International Research Consortium<br>Taasi , Geoffrey ; Uganda Ministry of Health<br>Barresi, Leah; Harvard T.H. Chan School of Public Health, Epidemiology<br>Bärnighausen, Till ; Heidelberg University, Institute of Public Health<br>Oldenburg, Catherine; University of California San Francisco, Francis I Proctor Foundation

Keywords:	HIV self-testing, female sex workers, accuracy, results interpretation, sensitivity, specificity

HIV self-testing process accuracy and results interpretation among female sex workers in Kampala, Uganda: a field assessment

Katrina Ortblad[1], Daniel Kibuuka Musoke[2], Thomson Ngabirano[3], Aidah Nakitende[2], Geoffrey Taasi[4], Leah G. Barresi[5], Till Bärnighausen[1,6,7], Catherine E. Oldenburg[8,9,10]

[1]Department of Global Health and Population, Harvard T.H. Chan School of Public Health, Boston, MA, USA
[2]International Research Consortium, Kampala, Uganda
[3]Uganda Health Marketing Group, Kampala, Uganda
[4]Ugandan Ministry of Health, Kampala, Uganda
[5]Department of Epidemiology, Harvard T.H. Chan School of Public Health, Boston, MA, USA
[6]Institute of Public Health, Faculty of Medicine, Heidelberg University, Heidelberg, Germany
[7]Africa Health Research Institute, KwaZulu-Natal, South Africa
[8]Francis I. Proctor Foundation, University of California, San Francisco, San Francisco, CA, USA
[9]Department of Ophthalmology, University of California, San Francisco, San Francisco, CA, USA
[10]Department of Epidemiology and Biostatistics, University of California, San Francisco, San Francisco, CA, USA

Journal: BMJ Open
Word count: 2,784
Abstract Word Count: 225
Tables: 2
Figures: 3
References: 38
Appendix Tables: 1

Corresponding Author:
Katrina F. Ortblad, MPH
Department of Global Health and Population
Harvard T.H. Chan School of Public Health
677 Huntington Ave
Boston, MA 02115
+1-206-265-1856
katrina.ortblad@mail.harvard.edu

Keywords: HIV self-testing; female sex workers; accuracy; results interpretation; sensitivity; specificity

For peer review only

ABSTRACT

Objectives: To evaluate HIV self-testing process accuracy and results interpretation among female sex workers (FSWs) in Kampala, Uganda in the absence of pre-test training.

Methods: In October 2016, 104 FSWs used an oral HIV self-test while under observation by research assistants. The FSWs were not given pre-test training on HIV self-test use, and were only given the manufacturer's pictorial and written instructions to guide them. Randomly drawn, anonymous, and used HIV self-tests were then interpreted by FSWs. We calculated FSW-interpreted HIV self-test sensitivity and specificity by comparing FSWs' interpretations of self-test results with investigators' interpretations of self-test results (derived from drawing by research assistants).

Results: Errors in the HIV self-testing process and misinterpretation of HIV self-test results were common among FSWs: 23% (12/56) of FSWs interpreted HIV-negative self-test results as HIV-positive and 8% (3/37) of FSWs interpreted HIV-positive self-test results as HIV-negative. FSW-interpreted HIV self-test sensitivity was 73% (95% CI 56-86%) and specificity was 68% (95% CI 54-80%).

Conclusion: In the absence of pre-test training, FSWs in Kampala, Uganda have difficulties correctly using HIV self-tests and interpreting the results. The FSW-interpreted HIV self-test sensitivity and specificity in this study was lower than that in measured in previous studies. Training on use and interpretation of HIV self-tests may be necessary to avoid the negative consequences of false positive and false negative HIV self-test results among FSWs.

For peer review only

For peer review only - http://bmjopen.bmj.com/site/about/guidelines.xhtml

ARTICLE SUMMARY

Strengths and limitations of this study

- This the first study to explore oral HIV self-test performance among Ugandan female sex workers in the absence of pre-test training; a realistic scenario where female sex workers might obtain self-test from pharmacies or friends.
- Unlike other studies, female sex workers in this study did not interpret their own HIV self-test result, thus their interpretations were not biased by knowledge of previous HIV test results or HIV risk encounters.
- Study limitations include our small sample size and our limited generalizability of study results to other populations of female sex workers.

For peer review only

INTRODUCTION

HIV self-testing has been shown to increase HIV testing compared to standard of care HIV testing services in diverse populations, [1–8] however, the benefits of HIV self-testing depend on the self-testers ability to correctly follow a sequence of steps, interpret self-test results, and know how to link to appropriate HIV prevention and treatment services. HIV self-testing might be particularly beneficial for female sex workers (FSWs), who are recommended to test for HIV frequently by the World Health Organization (WHO).[9] A recent randomized controlled health systems trial found that HIV self-testing significantly increased recent and repeat HIV testing compared to referral to standard HIV testing services among FSWs in Kampala, Uganda.[8]. FSWs, however, might have lower levels of health literacy [10] and educational attainment [11–13] compared to members of the general population, potentially resulting in greater difficulties with the HIV self-testing process, including self-test use and interpretation of self-test results.

Previous HIV self-test performance studies found high sensitivity and specificity of participant-interpreted HIV self-test results.[14–22]. In the majority of these studies, however, participants were provided with extensive pre-test training prior to HIV self-testing.[14–21] For example, in one study participants were asked to demonstrate their understanding of self-testing with a cotton bud and vial of water,[14], while in another study fisherfolk received a 10-minute demonstration on how to use the self-test prior to testing.[16] In a real world scenario, these pre-test training interventions would be difficult to implement with declining national HIV budgets and other competing health priorities.[23] Another limitation of the previous HIV self-test performance studies was that participants interpreted their own HIV self-test result.[14–22] When participants interpret their own self-test results, their interpretations are likely biased by knowledge of prior HIV test results and HIV risk encounters. To our knowledge, no study to date has measured HIV self-test performance among FSWs, an important population for HIV prevention interventions.[13,24]

For peer review only - http://bmjopen.bmj.com/site/about/guidelines.xhtml

In this study, we measure HIV self-test performance in the absence of any pre-test training among FSWs in Kampala, Uganda. The specific objectives of this study are to measure FSWs' ability to: (1) accurately complete the HIV testing process, (2) interpret HIV self-test results, and (3) understand the next steps for HIV treatment and prevention following hypothetical self-test results. Additionally, we measure FSWs' belief in the HIV self-test results, interest in future HIV self-testing, and willingness to pay for HIV self-testing. The results of this study will help inform real world implementation of HIV self-testing among members of a key population during a time when few sub-Saharan African countries have guidelines on HIV self-testing.[25]

METHODS

Ethics

The study was approved by the Institutional Review Boards at the Harvard T.H. Chan School of Public Health and the Mildmay Uganda Research Ethics Committee, it also received special permission from the Ugandan Ministry of Health to introduce a new HIV testing technology.

Setting

Kampala, the capitol city of Uganda, has roughly 13,000 FSWs operating in more than 180 venues. One in every three of these FSWs is estimated to be living with HIV.[26,27] The Ugandan Ministry of Health identifies FSWs as a priority population for HIV prevention interventions [28] and provides them with specialized health services (including clinics and community-based HIV testing) through the Most at Risk Populations Initiative (MARPI).

Participants

For peer review only - http://bmjopen.bmj.com/site/about/guidelines.xhtml

The FSWs in this study were recruited by established FSW peer organizations in Kampala or through MARPI team leaders. All the FSWs were participating in a two-day peer educator training for a HIV self-testing randomized controlled health systems trial.[8] A number of FSW had previous peer educator training, while others were being trained as peer educators for the first time. The two-day peer educator training included information on the role of the peer educator, HIV and STIs prevention, FSW-friendly HIV testing and treatment options in Kampala, and oral HIV self-testing. This study took place at the end of training day one, prior to any discussions around HIV testing options in Kampala or demonstrations of HIV self-testing. Participation in this study was voluntary, and FSWs were compensated 16,500 UGX (US $4.70) for their time. All FSWs provided written informed consent.

Process accuracy

The FSWs were silently observed HIV self-testing by research assistants. All FSWs used the OraQuick ADVANCE Rapid HIV-1/2 Antibody Test (OraSure Technologies, Bethlehem PA), an oral HIV self-test that delivers results in 20 minutes. Three to four FSWs were in a room at a time, and were spread out so they could not see or hear one another. The FSWs received no description of the oral HIV self-testing process or explanation of how to interpret the results prior to testing and they were instructed to not ask the research assistant any questions. All FSWs had access to written and pictorial OraQuick HIV self-test instructions, which included a 10-step guide on how to use the self-test as well as instructions on how to interpret the self-test results and link to care. The OraQuick instructions were available in both English and the local language, Luganda. Research assistants noted the time FSWs started and stopped the self-testing process and recorded the steps participants took on a standardized checklist based on the OraQuick instructions. The used HIV self-tests were taken to a private location while the results developed.

For peer review only

Result interpretation

FSWs interpreted a mock oral HIV self-test (a random, anonymous self-test used by a fellow participant) handed to them by a research assistant and completed a brief results questionnaire. The HIV self-test result reads like an at-home pregnancy test, where all valid results have at least one red band indicating the control. A single band indicates a nonreactive or negative test. Two red bands indicate a reactive or positive test. No red bands, blurred bands, or no control band indicate an invalid test result. The research assistant drew the results of the distributed mock self-test result on a standardized paper form and then asked the participants to interpret the mock self-test and describe the appropriate next steps in HIV treatment and prevention following hypothetical self-test results. The FSWs were also asked to specify their belief in the accuracy of the self-test, interest in future self-testing, and willingness to pay for HIV self-testing.

None of the FSWs in this study learned their HIV status as a result study participation and all self-tests were disposed of after study completion. The FSWs were not given their own self-test to interpret because many already knew their HIV status and this knowledge would bias our measurement of HIV self-test performance. We were additionally concerned about confidentiality of our participants in a group training environment. This procedure was reviewed and approved by all ethics boards. FSWs who wanted to know their status were referred to clinics in Kampala where they could access free HIV testing and counseling services.

Accuracy of oral HIVST

Accuracy of oral HIVST can be measured two distinct ways; the participant-interpreted HIV self-test results can be compared to a gold standard, such as laboratory blood-based test,[14,29,30] or an interpretation of the same test results by a trained individual. In this study we measured accuracy of HIV self-tests using the later of these two methods. FSW-interpreted HIV self-test results were compared

For peer review only - http://bmjopen.bmj.com/site/about/guidelines.xhtml

with interpretations of self-results by the investigators, derived from research assistant drawings of the mock self-tests. All self-test results with two bands (one control and one test) were considered positive, even if one of the bands was very light. All self-test results with only one control band were considered negative. All self-test results with no control band (even if a test band were present) were considered inconclusive.

Statistical Analysis

We calculated FSW-interpreted self-test sensitivity and specificity using FSWs' interpretations and investigators' interpretations (derived from research assistant drawings) of the mock HIV self-test results. Investigator-interpreted invalid self-test results were excluded from these calculations, while results interpreted as invalid or "don't know" by the FSWs were included in the denominator of these calculations. We used Stata 13.1 (Stata Corporation, College Station, TX) to estimate binomial 95% confidence intervals.

Participant and Public Involvement

The research question explored in this study was developed from conversations we had with the Ugandan Ministry of Health and was intended to address one of their concerns related to scaling HIV self-testing. A number of participants were additionally leaders of FSW peer organizations in Kampala and were consulted during the development of the study to gauge their interest in HIV self-testing. The leaders of FSW peer organizations also helped us identify and recruit other FSW peer educators for study participation and were invited to attend a national dissemination of study results.

RESULTS

For peer review only - http://bmjopen.bmj.com/site/about/guidelines.xhtml

In October 2016, 118 FSW participated in a two-day peer educator training for a HIV self-testing randomized controlled trial. Of these FSWs, 104 (88%) participated in HIV self-testing and 99 (84%) interpreted a mock HIV self-test and completed the results questionnaire (**Figure 1**). Conducting the oral HIV self-test took 7.02 minutes on average (standard deviation [sd] 4.75 minutes), of which 4.74 minutes on average were spent reading the oral HIV self-test instructions (sd 3.31 minutes). **Table 1** highlights the percentage of FSWs who completed specific, desirable steps in the testing process (in order of operation).

Table 1. FSWs' HIV self-test process accuracy; number of participants who completed task (n) and % of total participants observed (%), N=104

Category	n	% (95% CI)
1. Read instructions prior to testing	61	58% (49-68%)
2. Removed buffer cap	80	80% (68-85%)
3. Put buffer in buffer-stand	52	50% (40-60%)
4. Did not touch the swipe pad	73	70% (60-79%)
5. Swabbed upper gum	77	74% (65-82%)
6. Swabbed lower gum	72	69% (59-78%)
7. Put test stick in buffer	77	74% (65-82%)
8. Referenced instructions during testing	59	57% (47-66%)

Of 99 used HIV self-tests that were randomly distributed to FSWs for self-interpretation, 93 (94%) tests had valid results and 6 (6%) tests had invalid results (four had no result bands and two had no control band but a single test band). Of the valid tests, 56 (57%) had one control band and were considered HIV negative, while 37 (37%) had both a control and test band and were considered HIV positive (five of these had a weak test band and one had a weak control band).

Figure 2 illustrates how FSWs interpreted the mock oral HIV self-test they were distributed, by the investigators' interpretation of the test result (see **Appendix Table 1** for details). Of the 56 investigator-interpreted HIV-negative test results, FSWs correctly interpreted 38 as HIV-negative (68%, 95% CI 54-80%) and incorrectly interpreted 13 as HIV-positive or invalid (23%, 95% CI 13-36%). Of the 37

For peer review only - http://bmjopen.bmj.com/site/about/guidelines.xhtml

investigator-interpreted HIV-positive test results, FSWs correctly interpreted 27 as HIV-positive (73%, 95% CI 56-86%) and incorrectly interpreted three as HIV-negative (8%, 95% CI 2-22%). Only one of the six investigator-interpreted invalid test results was correctly interpreted as such by FSWs, the rest were incorrectly interpreted by FSWs as HIV-negative (83%, 95% CI 36-100%). These results translate into a FSW-interpreted self-test sensitivity of 73% (95% CI 56-86%) and specificity of 68% (95% CI 54-80%).

When FSWs were asked what the appropriate next steps for HIV treatment and prevention were following a specified HIV self-test result, few provided the responses suggested in the OraQuick instructions (**Table 2**). For example, only 25% (24/99) of FSWs said they would test again in 3 months following a HIV-negative test result, only 46% (45/99) of FSWs said they would get a confirmatory test to clinic following a HIV-positive test result, and only 58% (51/99) and 36% (32/99) of FSWs respectively said they would retake another oral HIV self-test or get confirmatory test at clinic following an invalid test result.

Table 2. Participant perceptions of the appropriate next steps following a specified oral HIV self-test result; number who reported each step (n) and percent of total participants (95% CI), N=99

	Specified self-test result					
	HIV-negative		*HIV-positive*		*Invalid*	
**Next step**	**n**	**%**	**n**	**%**	**n**	**%**
Test again in 12 months	0	0% (0-0%)	0	0% (0-0%)	0	0% (0-0%)
Test again in 3 months	24	25% (17-35%)	3	3% (1-8%)	4	5% (1-11%)
Retake another oral HIV self-test	8	8% (4-16%)	7	7% (3-14%)	51	58% (47-68%)
Get confirmatory test at clinic	20	21% (13-30%)	45	46% (36-56%)	32	36% (26-47%)
Don't know	2	2% (0-7%)	4	4% (1-10%)	3	3% (0-10%)
Other: Get counseling	-	-	21	21% (14-31%)	1	1% (0-6%)
Other: Start treatment	1	1% (0-6%)	41	42% (32-52%	2	2% (0-8%)
Other: Use protection	53	55% (44-65%)	4	4% (1-10%)	3	3% (1-10%)
Other: Look after yourself	1	1% (0-6%)	-	-	-	-
Other: Suicidal thoughts	-	-	1	1% (0-6%)	-	-
Other: Test again in 1.5 months	-	-	1	1% (0-6%)	-	-

For peer review only - http://bmjopen.bmj.com/site/about/guidelines.xhtml

Other: Consult others	-	-	-	-	2	2% (0-8%)
Other: Give up on testing	-	-	-	-	1	1% (0-6%)

Figure 3 shows that the majority of FSWs believed the HIV self-test results, were interested in HIV self-testing in the future, and were willing to pay for HIV self-testing. The price that the majority of FSWs (54%, 53/99) were willing to pay for an HIV self-test was 1000-5000 Ugandan Shillings (UGX) (US $0.3-$1.4); 23% (23/99) of FSWs reported a willingness to pay 5000-10000 UGX (US $1.4-$2.88) for an HIV self-test, and only 5% (5/99) of FSWs reported a willingness to pay greater than 10000 UGX (US $2.88) for an HIV self-test.

DISCUSSION

In the absence of pre-test training, FSWs in Uganda had difficulties following the steps in the HIV self-testing process, correctly interpreting the self-test results, and understanding the next appropriate steps for HIV treatment and prevention following HIV self-testing. The low FSW-interpreted HIV self-test sensitivity and specificity measurements in this study are concerning because false perception of HIV status may result in behaviors or contribute to mental states that are counterproductive to health. For example, an incorrectly perceived HIV-positive self-test result may lead to depression,[31] while an incorrectly perceived HIV-negative self-test result may delay HIV treatment. The low percentage of FSWs in this study that correctly identified the next steps for HIV treatment and prevention following hypothetical self-test results is also concerning because this suggests that even if FSWs correctly learn their HIV status by HIV self-testing, they may not know what behaviors or interventions can improve their health and prevent the transmission of HIV.[32,33]

The FSW-interpreted HIV self-test sensitivity and specificity measured in this study is significantly lower than that measured in other studies where participants were provided with pre-test training.[14–21] For example, participant-interpreted HIV self-test sensitivity was 93.4% and specificity

For peer review only - http://bmjopen.bmj.com/site/about/guidelines.xhtml

was 99.9% in a large Malawi study that provided pre-test training.[14] Understanding HIV self-test performance in the absence of pre-test training is important because this reflects a potential real-world scenario where FSWs purchase an HIV self-test from a pharmacy or receive one from a friend. Pre-test training interventions are also expensive, especially the intensity of those provided in the other HIV self-testing performance studies,[14–21] and thus difficult to implement at scale in the context of limited health budgets and competing HIV prevention interventions.[23] Our study also focused on FSWs, a key population that might have increased difficulties with HIV self-test performance because of different levels of health literacy and education compared to members of the general population.[24,34] Additionally, the FSWs in our study interpreted an HIV self-test result that was not their own, thus their interpretations were not biased by knowledge of previous HIV test results and past HIV risk encounters. Understanding how well FSWs can interpret HIV self-test results without bias of prior knowledge is important because HIV self-testing moves HIV testing outside the unregulated environment of the health system,[8] where FSWs might use an HIV self-test to test others (e.g., clients, children) or use an HIV self-test to test for HIV for the first time.

There are limitations with this study that are important to note. First, the sample size was small because this study was a sub-study of a larger HIV self-testing randomized controlled trial.[8] Second, the FSWs in this study were recommended by peer educators and not necessarily representative of the greater Kampala-based FSW population. The FSW peer educators in this study may have higher health literacy and education compared to other FSWs, potentially resulting in greater HIV self-test process accuracy and better interpretation of HIV self-test results. Third, the FSWs in this study were observed using the HIV self-tests instead of testing in isolation, which may have either positively or negatively influenced study results depending on if this made FSWs more nervous or careful while self-testing or interpreting self-test results [16]. Fourth, we had difficulty keeping FSWs separated from each other as

For peer review only - http://bmjopen.bmj.com/site/about/guidelines.xhtml

they waited to self-test and interpret self-test results. The FSWs may have discussed study procedures with one another, which could have improved their HIV self-test performance.

The findings from this study suggest that governments considering HIV self-testing need to carefully consider what information FSWs are given before they self-test and what resources will be available to them during or after self-testing. The intensity of pre-test training in previous HIV self-test performance studies may be difficult to scale and might not fit within existing national HIV budgets [23]. Simpler tools such as improved self-test instructions, self-test demonstration videos, or HIV self-testing counselors should be considered. Additionally, HIV self-test manufacturers should consider re-designing HIV self-tests to improve interpretation of self-test results. The cost for these additional tools will have to be considered on top of the already high costs of HIV self-tests, which we purchased at approximately $6 USD per self-test for research purposes in Uganda; a cost well above the $1.5 USD per self-test willingness to pay threshold expressed by the majority of FSWs in this study. In an era of stagnant international aid dedicated to HIV [23], it is important to consider the cost and benefits of HIV self-testing in relation to other HIV testing interventions [35–38].

CONCLUSIONS

Distributing oral HIV self-tests to Kampala-based FSWs without any pre-test training can result in errors in the self-testing process, incorrect interpretations of self-test results, and incorrect perceptions about the appropriate next steps for HIV treatment and prevention following HIV self-testing. Despite challenges using and interpreting HIV self-tests, the majority of FSWs in this study believed the self-test results and were interested and willing to pay for HIV self-tests in the future. The results from this study demonstrate that FSWs are enthusiastic about HIV self-testing, but highlight the importance of pre-test training or other support interventions for HIV self-testing to prevent mistakes in the self-testing process, misperceptions about HIV status, and delays in HIV treatment or prevention interventions.

For peer review only - http://bmjopen.bmj.com/site/about/guidelines.xhtml

ACKNOWLEDGMENTS

We would like to acknowledge all the FSWs who took time participating in this study as well as the research assistants who took time collecting this data.

FUNDING STATEMENT

This study was funded by the International Initiative for Impact Evaluation (3ie). KFO was additionally supported by the National Institute of Allergy and Infectious Disease T32-AI007535 (PI: Seage). CEO was supported in part by the National Institute on Drug Abuse T32-DA013911 (PI: Flanigan) and the National Institute of Allergy and Mental Health R25-MH083620 (PI: Nunn). TB was funded by the Alexander von Humboldt Foundation through the Alexander von Humboldt Professorship endowed by the German Federal Ministry of Education and Research. He was also supported by the Wellcome Trust, the European Commission, the Clinton Health Access Initiative and NICHD of NIH R01-HD084233, NIAID of NIH R01-AI124389 and R01-AI112339 and FIC of NIH D43-TW009775. The funders had no role in study design, data collection and analysis, decision to publish, or preparation of the manuscript.

COMPETING INTEREST STATEMENT

The authors of this study declare no conflicts of interest.

INC. CONTRI. STATEMENT

KFO, DKM and CEO designed the study and conceptualized the paper. KFO conducted the analysis and wrote the first draft. KFO, CEO, TN, DKM, GT, LB and AN oversaw the collection and the quality data. CEO and TB supervised the study. KFO, DKM, TN, AN, GT, LB, TB and CEO all edited and revised the manuscript.

DATA SHARING STATEMENT

Data is available from the authors upon request.

For peer review only

REFERENCES

1 Ayles H, Floyd S, Mulubwa C, *et al.* Increasing knowledge of HIV status among men: a cluster-randomised trial of community-based distribution of oral HIV self-test kits nested in four HPTN 071 communities in Zambia. Paris, France: 2017. http://programme.ias2017.org/Abstract/Abstract/5600

2 Gichangi A, Wambua J, Gohole A, *et al.* Provision of oral HIV self-test kits triples uptake of HIV testing among male partners of antenatal care clients: results of a randomized trial in Kenya. Durban, South Afirca: 2016.

3 Masters SH, Agot K, Obonyo B, *et al.* Promoting Partner Testing and Couples Testing through Secondary Distribution of HIV Self-Tests: A Randomized Clinical Trial. *PLOS Med* 2016;**13**:e1002166. doi:10.1371/journal.pmed.1002166

4 Thirumurthy H, Masters SH, Mavedzenge SN, *et al.* Promoting male partner HIV testing and safer sexual decision making through secondary distribution of self-tests by HIV-negative female sex workers and women receiving antenatal and post-partum care in Kenya: a cohort study. *Lancet HIV* 2016;**3**:e266-274. doi:10.1016/S2352-3018(16)00041-2

5 Johnson CC, Kennedy C, Fonner V, *et al.* Examining the effects of HIV self-testing compared to standard HIV testing services: a systematic review and meta-analysis. *J Int AIDS Soc* 2017;**20**. doi:10.7448/ias.20.1.21594

6 Wang Z, Lau JTF, Ip M, *et al.* A Randomized Controlled Trial Evaluating Efficacy of Promoting a Home-Based HIV Self-Testing with Online Counseling on Increasing HIV Testing Among Men Who Have Sex with Men. *AIDS Behav* Published Online First: 22 August 2017. doi:10.1007/s10461-017-1887-2

For peer review only - http://bmjopen.bmj.com/site/about/guidelines.xhtml

7 Katz D, Golden M, Hughes J, *et al.* HIV self-testing increases HIV testing frequency among high-risk men who have sex with men: a randomized controlled trial. Vancouver, Canada:

8 Ortblad K, Kibuuka Musoke D, Ngabirano T, *et al.* Direct provision versus facility collection of HIV self-tests among female sex workers in Uganda: a cluster-randomized controlled health systems trial. *PLoS Med* 2017;**14**:e1002458.

9 Consolidated Guidelines on HIV Testing Services; 5Cs: Consent, Confidentiality, Counseling, Correct Results and Connection. Geneva, Switzerland: : WHO 2015. http://apps.who.int/iris/bitstream/10665/179870/1/9789241508926_eng.pdf

10 Parker RG, Perez-Brumer A, Garcia J, *et al.* Prevention literacy: community-based advocacy for access and ownership of the HIV prevention toolkit. *J Int AIDS Soc* 2016;**19**. doi:10.7448/IAS.19.1.21092

11 Scorgie F, Chersich MF, Ntaganira I, *et al.* Socio-demographic characteristics and behavioral risk factors of female sex workers in sub-saharan Africa: a systematic review. *AIDS Behav* 2012;**16**:920–33. doi:10.1007/s10461-011-9985-z

12 Ngugi EN, Roth E, Mastin T, *et al.* Female sex workers in Africa: epidemiology overview, data gaps, ways forward. *SAHARA J J Soc Asp HIVAIDS Res Alliance* 2012;**9**:148–53. doi:10.1080/17290376.2012.743825

13 Shannon K, Strathdee SA, Goldenberg SM, *et al.* Global epidemiology of HIV among female sex workers: influence of structural determinants. *Lancet Lond Engl* 2015;**385**:55–71. doi:10.1016/S0140-6736(14)60931-4

For peer review only

For peer review only - http://bmjopen.bmj.com/site/about/guidelines.xhtml

14 Choko AT, MacPherson P, Webb EL, *et al.* Uptake, Accuracy, Safety, and Linkage into Care over Two Years of Promoting Annual Self-Testing for HIV in Blantyre, Malawi: A Community-Based Prospective Study. *PLoS Med* 2015;**12**:e1001873. doi:10.1371/journal.pmed.1001873

15 Choko AT, Desmond N, Webb EL, *et al.* The Uptake and Accuracy of Oral Kits for HIV Self-Testing in High HIV Prevalence Setting: A Cross-Sectional Feasibility Study in Blantyre, Malawi. *PLOS Med* 2011;**8**:e1001102. doi:10.1371/journal.pmed.1001102

16 Asiimwe S, Oloya J, Song X, *et al.* Accuracy of un-supervised versus provider-supervised self-administered HIV testing in Uganda: A randomized implementation trial. *AIDS Behav* 2014;**18**:2477–84. doi:10.1007/s10461-014-0765-4

17 Kurth AE, Cleland CM, Chhun N, *et al.* Accuracy and Acceptability of Oral Fluid HIV Self-Testing in a General Adult Population in Kenya. *AIDS Behav* 2016;**20**:870–9. doi:10.1007/s10461-015-1213-9

18 Pai NP, Behlim T, Abrahams L, *et al.* Will an Unsupervised Self-Testing Strategy for HIV Work in Health Care Workers of South Africa? A Cross Sectional Pilot Feasibility Study. *PLOS ONE* 2013;**8**:e79772. doi:10.1371/journal.pone.0079772

19 Dong M, Regina R, Hlongwane S, *et al.* Can laypersons in highprevalence South Africa perform an HIV sefl-test accurately? Melbourne, Australia: 2014.

20 Napierala Mavedzenge S, Sibanda E, Mavengere Y, *et al.* Supervised HIV self-testing to inform implementation and scale up of self-testing in Zimbabwe. *J Int AIDS Soc* Published Online First: 1 July 2015.https://www.rti.org/publication/supervised-hiv-self-testing-inform-implementation-and-scale-self-testing-zimbabwe (accessed 20 Mar 2017).

For peer review only

21 Martínez Pérez G, Steele SJ, Govender I, *et al.* Supervised oral HIV self-testing is accurate in rural KwaZulu-Natal, South Africa. *Trop Med Int Health TM IH* 2016;**21**:759–67. doi:10.1111/tmi.12703

22 Figueroa C, Johnson C, Ford N, *et al.* Reliability of HIV Rapid Diagnostic Tests for Self-Testing Performed by Self-Testers Compared to Healthcare Workers: A Systematic Review and Meta-Analysis. *Rev* 2017.

23 Financing Global health 2015: Development assistance steady on the path to new Global Goals. Seattle, WA: : Insitution for Health Metrics and Evalution 2016. http://www.healthdata.org/policy-report/financing-global-health-2015-development-assistance-steady-path-new-global-goals (accessed 28 Mar 2017).

24 Baral S, Beyrer C, Muessig K, *et al.* Burden of HIV among female sex workers in low-income and middle-income countries: a systematic review and meta-analysis. *Lancet Infect Dis* 2012;**12**:538–49. doi:10.1016/S1473-3099(12)70066-X

25 Policy & Regulations for HIVST. HIVST.org. http://www.hivst.org/policy-regulations-for-hivst-1/ (accessed 8 Sep 2015).

26 Crane Survey: Select Results from Recent Surveys, Kampala 2012/3. http://documentslide.com/documents/crane-survey-select-results-from-recent-surveys-kampala-20123-men-who-have.html (accessed 28 Mar 2017).

27 HIV and AIDS Uganda Country Progress Report; 2013. Kampala, Uganda: : Uganda AIDS Comission 2014. http://www.uhasselt.be/Documents/UHasselt/onderwijs/internationaal/noord-zuid_2015/HIV_and_AIDS_Uganda_Country_Progress_Report_2013.pdf (accessed 5 Sep 2015).

For peer review only

For peer review only - http://bmjopen.bmj.com/site/about/guidelines.xhtml

28 National HIV Testing Services Policy and Implementation Guidelines Uganda: 4th Edition. Kampala, Uganda: Ministry of Health 2016.

29 OraQuick In-Home HIV Test; Summary of Safety and Effectiveness. US Food and Drug Administration 2012. https://www.fda.gov/downloads/BiologicsBloodVaccines/BloodBloodProducts/ApprovedProducts/PremarketApprovalsPMAs/UCM312534.pdf

30 Figeroa C, Johnson C, Deivanayagam T, *et al.* Considerations on the accuracy and reliability of HIV self-testing: a literature review.

31 L'akoa RM, Noubiap JJN, Fang Y, *et al.* Prevalence and correlates of depressive symptoms in HIV-positive patients: a cross-sectional study among newly diagnosed patients in Yaoundé, Cameroon. *BMC Psychiatry* 2013;**13**:228. doi:10.1186/1471-244X-13-228

32 Cohen MS, Chen YQ, McCauley M, *et al.* Prevention of HIV-1 Infection with Early Antiretroviral Therapy. *N Engl J Med* 2011;**365**:493–505. doi:10.1056/NEJMoa1105243

33 Donnell D, Baeten JM, Kiarie J, *et al.* Heterosexual HIV-1 transmission after initiation of antiretroviral therapy: a prospective cohort analysis. *The Lancet* 2010;**375**:2092–8. doi:10.1016/S0140-6736(10)60705-2

34 The Crane Survey: Female sex workers (FSW) in Kampala. Kampala, Uganda: : CDC 2009. https://docs.google.com/viewer?a=v&pid=sites&srcid=ZGVmYXVsdGRvbWFpbnxjcmFuZXN1cnZleXxneDo3NzE4MjNjM2YwYzRkY2E4 (accessed 28 Mar 2017).

For peer review only

35 Ending AIDS: Progress Towards the 90-90-90 Targets. Geneva, Switzerland: : UNAIDS 2017. http://www.unaids.org/sites/default/files/media_asset/Global_AIDS_update_2017_en.pdf (accessed 1 Aug 2017).

36 Sweat M, Morin S, Celentano D, *et al.* Community-based intervention to increase HIV testing and case detection in people aged 16–32 years in Tanzania, Zimbabwe, and Thailand (NIMH Project Accept, HPTN 043): a randomised study. *Lancet Infect Dis* 2011;**11**:525–32. doi:10.1016/S1473-3099(11)70060-3

37 Lugada E, Levin J, Abang B, *et al.* Comparison of home and clinic-based HIV testing among household members of persons taking antiretroviral therapy in Uganda: results from a randomized trial. *J Acquir Immune Defic Syndr 1999* 2010;**55**:245–52. doi:10.1097/QAI.0b013e3181e9e069

38 Iwuji CC, Orne-Gliemann J, Larmarange J, *et al.* Uptake of Home-Based HIV Testing, Linkage to Care, and Community Attitudes about ART in Rural KwaZulu-Natal, South Africa: Descriptive Results from the First Phase of the ANRS 12249 TasP Cluster-Randomised Trial. *PLOS Med* 2016;**13**:e1002107. doi:10.1371/journal.pmed.1002107

For peer review only

FIGURE LEGENDS

Figure 1. Flow of study procedures and number of participants in analysis populations

Figure 2. FSWs' interpretations of the HIV self-test results, by investigator interpretation of the same self-test (derived from a research assistant drawing)

Figure 3. FSWs' belief in the HIV self-test results, future interest in HIV self-testing, and willingness to pay for an HIV self-test in the future

For peer review only

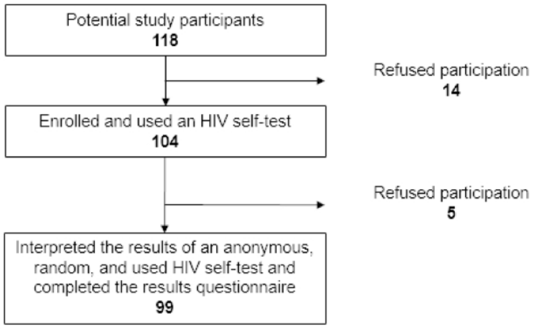

Figure 1. Flow of study procedures and number of participants in analysis populations

279x361mm (300 x 300 DPI)

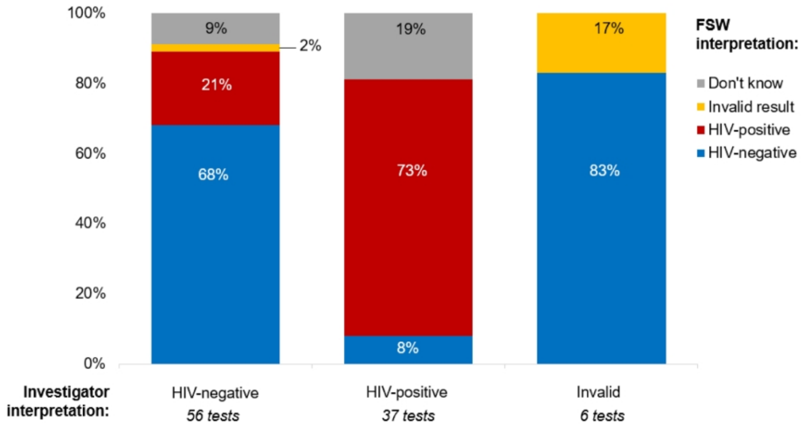

Figure 2. FSWs' interpretations of the HIV self-test results, by investigator interpretation of the same self-test (derived from a research assistant drawing)

215x166mm (300 x 300 DPI)

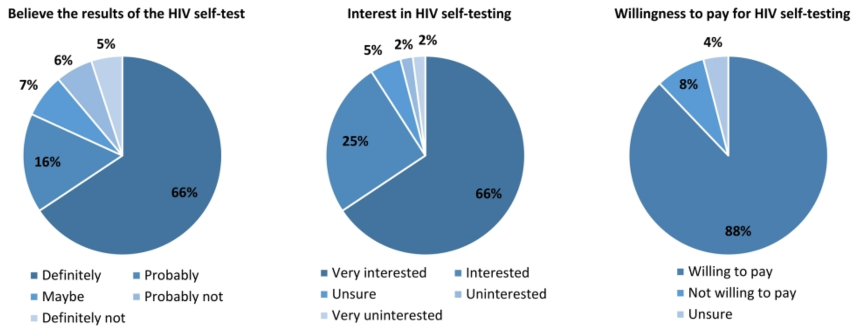

Figure 3. FSWs' belief in the HIV self-test results, future interest in HIV self-testing, and willingness to pay for an HIV self-test in the future

215x166mm (300 x 300 DPI)

Appendix Table 1. FSW-interpretation of the HIV self-test results compared to investigator-interpretation of the same results; DK=don't know.

		Investigator interpretation			
		HIV-positive	*HIV-negative*	*Invalid/DK*	**Total**
**FSW interpretation**	*HIV-positive*	27	12	0	39
	HIV-negative	3	38	5	46
	Invalid/DK	7	6	1	14
	**Total**	37	56	6	99

For peer review only

BMJ Open

HIV self-test performance among female sex workers in Kampala, Uganda: a cross-sectional study

Journal:	*BMJ Open*
Manuscript ID	bmjopen-2018-022652.R1
Article Type:	Research
Date Submitted by the Author:	23-May-2018
Complete List of Authors:	Ortblad, Katrina; Harvard T.H. Chan School of Public Health, Global Health and Population<br>Kibuuka Musoke , Daniel ; International Research Consortium<br>Ngabirano , Thomson ; Uganda Health Marketing Group<br>Nakitende, Aidah ; International Research Consortium<br>Taasi , Geoffrey ; Uganda Ministry of Health<br>Barresi, Leah; Harvard T.H. Chan School of Public Health, Epidemiology<br>Bärnighausen, Till ; Heidelberg University, Institute of Public Health<br>Oldenburg, Catherine; University of California San Francisco, Francis I Proctor Foundation
**Primary Subject Heading**:	HIV/AIDS
Secondary Subject Heading:	Global health, Epidemiology, Public health
Keywords:	HIV self-testing, female sex workers, sensitivity, specificity, Uganda, Africa

For peer review only

SCHOLARONE™
Manuscripts

HIV self-test performance among female sex workers in Kampala, Uganda: a cross-sectional study

Katrina Ortblad[1], Daniel Kibuuka Musoke[2], Thomson Ngabirano[3], Aidah Nakitende[2], Geoffrey Taasi[4], Leah G. Barresi[5], Till Bärnighausen[1,6,7], Catherine E. Oldenburg[8,9,10]

[1]Department of Global Health and Population, Harvard T.H. Chan School of Public Health, Boston, MA, USA
[2]International Research Consortium, Kampala, Uganda
[3]Uganda Health Marketing Group, Kampala, Uganda
[4]Ugandan Ministry of Health, Kampala, Uganda
[5]Department of Epidemiology, Harvard T.H. Chan School of Public Health, Boston, MA, USA
[6]Institute of Public Health, Faculty of Medicine, Heidelberg University, Heidelberg, Germany
[7]Africa Health Research Institute, KwaZulu-Natal, South Africa
[8]Francis I. Proctor Foundation, University of California, San Francisco, San Francisco, CA, USA
[9]Department of Ophthalmology, University of California, San Francisco, San Francisco, CA, USA
[10]Department of Epidemiology and Biostatistics, University of California, San Francisco, San Francisco, CA, USA

Journal: BMJ Open
Word count: 2,784
Abstract Word Count: 225
Tables: 2
Figures: 3
References: 38
Appendix Tables: 1

Corresponding Author:
Katrina F. Ortblad, MPH
Department of Global Health and Population
Harvard T.H. Chan School of Public Health
677 Huntington Ave
Boston, MA 02115
+1-206-265-1856
katrina.ortblad@mail.harvard.edu

Keywords: HIV self-testing; female sex workers; accuracy; results interpretation; sensitivity; specificity; Africa; Uganda

For peer review only

ABSTRACT

Objective: To evaluate HIV self-testing performance and results interpretation among female sex workers (FSWs) in Kampala, Uganda who did not receive pre-test training.

Methods: In October 2016, 104 participants used an oral HIV self-test while under observation by research assistants. Participants were not given pre-test training on HIV self-test use, and were only given the manufacturer's pictorial and written instructions to guide them. Research assistants recorded if participants completed and/or had difficulties with steps in the HIV self-testing process on a pre-specified checklist. Randomly drawn, used HIV self-tests were interpreted by FSWs. We calculated FSW-interpreted HIV self-test sensitivity and specificity by comparing FSWs' interpretations of self-test results with those indicated in the manufacturer's instruction guide.

Results: Only 33% (34/104) of participants completed all of the key steps in the HIV self-testing process and the majority (86%, 89/104) were observed having difficulties with at least one of these steps. Misinterpretation of HIV self-test results were common among FSWs: 23% (12/56) of FSWs interpreted HIV-negative self-test results as HIV-positive and 8% (3/37) of FSWs interpreted HIV-positive self-test results as HIV-negative. FSW-interpreted HIV self-test sensitivity was 73% (95%CI 56-86%) and specificity was 68% (95%CI 54-80%).

Conclusions: FSWs in Kampala, who did not receive any pre-test training, skipped steps in the HIV self-testing process and had difficulties correctly interpreting self-test results. The FSW-interpreted HIV self-test sensitivity and specificity in this study was lower than that in measured in previous studies. Training on use and interpretation of HIV self-tests may be necessary to prevent errors in the HIV self-testing process and to avoid the negative consequences of false positive and false negative HIV self-test results among FSWs.

For peer review only - http://bmjopen.bmj.com/site/about/guidelines.xhtml

ARTICLE SUMMARY

Strengths and limitations of this study

- This is one of few studies to explore oral HIV self-test performance among female sex workers in the absence of pre-test training; a realistic scenario where female sex workers might obtain HIV self-tests from pharmacies or friends.
- Unlike other studies, female sex workers in this study did not interpret their own HIV self-test result, thus their interpretations were not biased by knowledge of previous HIV test results or HIV risk encounters.
- Study limitations include our small sample size and our limited generalizability of study results to other populations of female sex workers.

For peer review only

INTRODUCTION

HIV self-testing has been shown to increase HIV testing compared to standard of care HIV testing services in diverse populations,[1–8] however, the benefits of HIV self-testing depend on the self-testers ability to correctly follow a sequence of steps, interpret self-test results, and know how to link to appropriate HIV prevention and treatment services. HIV self-testing might be particularly beneficial for female sex workers (FSWs), who are recommended to test for HIV frequently by the World Health Organization (WHO).[9] A recent randomized controlled health systems trial found that HIV self-testing significantly increased recent and repeat HIV testing compared to referral to standard HIV testing services among FSWs in Kampala, Uganda.[8] FSWs, however, often have lower levels of health literacy [10] and educational attainment [11–13] compared to members of the general population, potentially resulting in greater difficulties with the HIV self-testing process, including self-test use and interpretation of self-test results.

Previous HIV self-test performance studies found high sensitivity and specificity of participant-interpreted HIV self-test results.[14–23] In the majority of these studies, however, participants were provided with extensive pre-test training prior to HIV self-testing.[14–16,18,21] For example, in one study participants were asked to demonstrate their understanding of self-testing with a cotton bud and vial of water,[14] while in another study, fisherfolk received a 10-minute demonstration on how to use the self-test prior to testing.[16] In a real world scenario, these pre-test training interventions would be difficult to implement with declining national HIV budgets and other competing health priorities.[24] In previous HIV self-test performance studies participants interpreted their own HIV self-test result, potentially introducing bias attributable to knowledge of prior HIV test results and HIV risk encounters.[14–22] Understanding how well individuals can interpret HIV self-test results without bias of prior knowledge is important because as HIV self-testing moves HIV testing outside the unregulated environment of the health system,[8] individuals might use an HIV self-test to test others (e.g., sexual

For peer review only - http://bmjopen.bmj.com/site/about/guidelines.xhtml

partners, children) or use an HIV self-test for first time HIV testing. Few studies to date has measured HIV self-test performance among FSWs,[22,25] an important population for HIV prevention interventions.[13,26]

In this study, we measured HIV self-test performance in the absence of any pre-test training among FSWs in Kampala, Uganda. Specifically, we explore the feasibility and usability of HIV self-tests among FSWs, including FSWs' ability to complete the sequence of steps necessary for self-testing, interpret HIV self-test results, and identify the next steps for HIV treatment and prevention. Additionally, we explore FSWs' values and preferences for HIV self-test, including acceptability and willingness to pay for HIV self-testing. The results of this study are intended to inform real world implementation of HIV self-testing among members of a key population during a time when few sub-Saharan African countries have guidelines on HIV self-testing.[27]

METHODS

Ethics

The study was approved by the Institutional Review Boards at the Harvard T.H. Chan School of Public Health and the Mildmay Uganda Research Ethics Committee, it also received special permission from the Ugandan Ministry of Health to introduce a new HIV testing technology.

Setting

Kampala, the capitol city of Uganda, has roughly 13,000 FSWs operating in more than 180 venues. One in every three of these FSWs is estimated to be living with HIV.[28,29] The Ugandan Ministry of Health identifies FSWs as a priority population for HIV prevention interventions [30] and provides them with specialized health services (including clinics and community-based HIV testing) through the Most at Risk Populations Initiative (MARPI).

For peer review only

Participants

The participants in this study were in training to be peer educators for an HIV self-testing randomized controlled health systems trial among FSWs.[8] We determined the number of peer educators necessary (120) based on our sample and cluster size for the main trial.[8] To recruit FSW peer educators for the trial, we used established FSW peer organizations in Kampala and MARPI team leaders. Peer educators were eligible for participation if they were 18 years or older and were accepted among members of the FSW community. The peer educator training was two days: day one covered trial procedures, information on the role of the peer educator, and HIV and STIs prevention, while day two covered FSW-friendly HIV testing and treatment options in Kampala and oral HIV self-testing (including how to perform the HIV self-test and interpret the test results). This study took place at the end of training day one, prior to any demonstrations of HIV self-testing. Participation in this study was voluntary, and participants were compensated 16,500 UGX (US $4.70) for their time. All participants provided written informed consent in the language in which they were most comfortable. Those who could not write provided consent with a thumbprint.

Usability of HIV self-tests

The participants were silently observed HIV self-testing by research assistants. All participants used the OraQuick ADVANCE Rapid HIV-1/2 Antibody Test (OraSure Technologies, Bethlehem PA), an oral HIV self-test that delivers results in 20 minutes. Testing took place in large, open rooms that were being used for peer educator training. Three to four participants were in a room at a time and spread out (approximately 10 meters apart) so they could not see or hear one another. The participants received no description of the HIV self-testing process or explanation of how to interpret the results prior to testing and were instructed to not ask the research assistant any questions. All participants had

For peer review only - http://bmjopen.bmj.com/site/about/guidelines.xhtml

access to written and pictorial OraQuick HIV self-test instructions (available in both English and the local language, Luganda), which included a guide on how to use the self-test as well as instructions on how to interpret the self-test results and link to care. We did not adapt this guide for use in this study.

Research assistants recorded the steps participants took when using the HIV self-testing on a standardized checklist, based on the OraQuick instructions. The checklist included: (1) removed buffer cap, (2) put buffer in buffer-stand, (3) swapped upper or lower gum, and (4) put test stick in buffer. Research assistants were instructed to indicate if participants had difficulties with any of these HIV self-testing steps. Additionally, research assistants recorded if participants read the HIV self-test instructions and when they read these instructions. Only one research assistant observed each participant as they HIV self-tested. All used HIV self-tests were taken to a back room (where no participants were present) by the study coordinator so that the results could process in private.

Interpretation of HIV self-test results

Once the HIV self-test results had processed, these self-tests were placed in a large opaque bag by the study coordinator. Research assistants drew a random HIV self-test from this bag for participants to interpret. All valid OraQuick results have at least one red band indicating the control. According to the OraQuick HIV self-testing instruction guide, a single band indicates a nonreactive or negative test, two red bands indicate a reactive or positive test, and no red bands, blurred bands, or no control band indicate an invalid test. Research assistants recorded the results of the HIV self-test they selected by drawing one line, two lines, no lines, or blurred lines on a standardized paper form before handing the HIV self-test to a participant for interpretation. All participants again received the manufacturer's written and pictorial instructions to aid their interpretations.

None of the participants in this study learned their HIV status as a result study participation and all self-tests were disposed of after study completion. The participants were not given their own self-test

For peer review only - http://bmjopen.bmj.com/site/about/guidelines.xhtml

to interpret because many already knew their HIV status and this knowledge would bias our measurement of HIV self-test performance. We were additionally concerned about confidentiality of our participants in a group training environment. Participants who wanted to know their status were referred to clinics in Kampala where they could access free HIV testing and counseling services.

Perceptions of HIV self-testing

All participants completed a brief questionnaire after interpreting a random HIV self-test result. This questionnaire asked participants to describe the appropriate next steps in HIV treatment and prevention following hypothetical self-test results. The questionnaire also asked participants to specify their belief in the accuracy of the self-test results, interest in future self-testing, and willingness to pay for HIV self-testing.

Statistical analysis

We calculated the proportion of participants that completed steps in the HIV self-testing process and the proportion of participants that had difficulties with each of these steps. To measure FSW-interpreted HIV self-test sensitivity and specificity, we calculated the proportion of participants that correctly interpreted the strong HIV-positive (two clear bands) and strong HIV-negative (one clear band) self-test results. The 'correct interpretation' of the self-test result was determined from the research assistant drawings of the self-test results, which were interpreted as indicated in the manufacturer's instruction guide. We excluded invalid HIV self-test and weak HIV-positive self-test results from our FSW-interpreted HIV self-test sensitivity and specificity calculations because these testing outcomes are less common and we wanted to focus on how well participants could interpret clear self-test results. We did not compare FSW-interpreted HIV self-test results with laboratory blood-based HIV test results, because when interpreted correctly by trained users, oral HIV self-testing has >99% sensitivity and

For peer review only - http://bmjopen.bmj.com/site/about/guidelines.xhtml

specificity.[31–33] Stata 13.1 (Stata Corporation, College Station, TX) was used to estimate binomial 95% confidence intervals for these measurements. We then calculated the proportion of participants that described their various perceptions of HIV self-testing, including the next steps following HIV self-test results and accuracy of the HIV self-tests.

Participant and public involvement

The research question explored in this study was developed from conversations we had with the Ugandan Ministry of Health and was intended to address one of their concerns related to scaling HIV self-testing. A number of participants were additionally leaders of FSW peer organizations in Kampala and were consulted during the development of the study to gauge their interest in HIV self-testing. These leaders of FSW peer organizations also helped recruitment of study participants and were invited to attend a national dissemination of study results.

For peer review only

RESULTS

In October 2016, 118 FSWs in urban Kampala, Uganda completed day one of the two-day peer educator training. Of these, 104 (88%) agreed to perform an HIV self-test and 99 (84%) interpreted a HIV self-test and completed the questionnaire. Fourteen of the FSWs who participated in day one of the two-day peer educator training refused to perform HIV self-testing and five of the participants who performed HIV self-testing refused to interpret an HIV self-test result.

Table 1 describes socio-demographic characteristics of study participants. The median age of study participants was 33 years (interquartile range: 29 to 27 years). The majority of participants (59%) reported completing some level of secondary education (9 to 12 years) and 90% of participants reported the ability to read and write. In Uganda, all public schooling on reading and writing is conducted in English. Almost all participants (83%) had worked as a peer educator at least once before participating in

For peer review only - http://bmjopen.bmj.com/site/about/guidelines.xhtml

this study, 59% of which had previously worked for Uganda's Most at Risk Population Initiative (also known as MARPI), which we used to help recruit peer educators for this study.

Table 1. Socio-demographic characteristics of study participants. N=104

Characteristics	N (%)
Age (median, IQR)	33 (29 to 37)
Education	
No formal (0 years)	4 (4%)
Primary/Junior (<9 years)	28 (27%)
Secondary (9-12 years)	61 (59%)
Vocational	5 (5%)
Tertiary	6 (6%)
Can read and write	94 (90%)
Previously worked as a FSW peer educator	86 (83%)
Times works as a peer educator (median, IQR)	2 (1 to 4)

Table 2 highlights the percentage of participants who completed and had difficulties with steps in the HIV self-testing process. While the majority of participants completed each of the individual steps (80% removed the buffer cap; 50% put the buffer in the buffer stand; 80% swabbed the upper or lower gum; 74% put the test stick in the buffer), only 33% of participants completed all four of these steps and the majority of participants (86%) were observed by research assistants as having difficulties with at least one of the steps. The majority of participants read the manufacturer's self-test instructions prior to testing (58%) or during the HIV self-testing process (57%), but 49% were observed as having difficulties with the instructions.

Table 2. The percentage of participants that completed and had difficulties with steps in the HIV self-testing process. N=104*

For peer review only

Step	Completed N (%)	Had difficulty N (%)
1. Removed buffer cap	80 (80%)	39 (38%)
2. Put buffer in buffer-stand	52 (50%)	72 (69%)
3. Swabbed upper or lower gum	82 (80%)	53 (51%)
4. Put test stick in buffer	77 (74%)	35 (34%)
All steps 1-4[1]	34 (33%)	14 (13%)
Any step 1-4[2]	102 (98%)	89 (86%)
Read instructions prior to testing	61 (58%)	51 (49%)[3]
Referenced instructions during testing	59 (57%)	

*Denominators vary based on completeness in reporting

[1]Participants were observed completing all steps 1-4 or having difficulties with all steps 1-4.

[2]Participants were observed completing any of the steps 1-4 or having difficulties with any of the steps 1-4.

[3]Participants were observed having any difficulties with reading the instructions.

Of 99 used HIV self-tests that were randomly distributed to participants for self-interpretation, 93 (94%) tests had valid results and 6 (6%) tests had invalid results (four had no result bands and two had no control band but a single test band). Of the valid tests, 56 (57%) had one control band and were considered HIV-negative, while 37 (37%) had both a control and test band and were considered HIV-positive (five of these had a weak test band and one had a weak control band).

Figure 1 illustrates how participants interpreted the HIV self-test they were distributed, by how the self-test should have been interpreted according to the OraQuick instruction guide. Of the 56 HIV-negative self-test results, participants correctly interpreted 38 (68%) as HIV-negative and incorrectly interpreted 13 (23%) as HIV-positive or invalid. Of the 37 HIV-positive self-test results, participants correctly interpreted 27 (73%) as HIV-positive and incorrectly interpreted three as HIV-negative (8%). Only one of the invalid self-test results was correctly interpreted as such by participants, the rest were

For peer review only

incorrectly interpreted by participants as HIV-negative (83%). These results translate into a FSW-interpreted self-test sensitivity of 73% (95% CI 56-86%) and specificity of 68% (95% CI 54-80%).

When participants were asked what the appropriate next steps for HIV treatment and prevention were following a specified HIV self-test result, few provided the responses suggested in the OraQuick instructions (**Table 3**). For example, only 25% (24/99) of participants said they would test again in 3 months following a HIV-negative test result, only 46% (45/99) of participants said they would get a confirmatory test to clinic following a HIV-positive test result, and only 58% (51/99) and 36% (32/99) of participants respectively said they would retake another oral HIV self-test or get confirmatory test at clinic following an invalid test result.

Table 3. Participant perceptions of the next steps following HIV self-test results. N=99

	**HIV self-test result**		
**Next step**	***HIV-negative*** **n (%)**	***HIV-positive*** **n (%)**	***Invalid*** **n (%)**
Test again in 12 months	0 (0%)	0 (0%)	0 (0%)
Test again in 3 months	24 (25%)	3 (3%)	4 (5%)
Retake another oral HIV self-test	8 (8%)	7 (7%)	51 (58%)
Get confirmatory test at clinic	20 (21%)	45 (46%)	32 (36%)
Don't know	2 (2%)	4 (4%)	3 (3%)
Other: Get counseling	-	21 (21%)	1 (1%)
Other: Start treatment	1 (1%)	41 (42%)	2 (2%)
Other: Use protection	53 (53%)	4 (4%)	3 (3%)
Other: Look after yourself	1 (1%)	-	-
Other: Suicidal thoughts	-	1 (1%)	-
Other: Test again in 1.5 months	-	1 (1%)	-
Other: Consult others	-	-	2 (2%)

For peer review only - http://bmjopen.bmj.com/site/about/guidelines.xhtml

Other: Give up on testing	-	-	1 (1%)

Figure 2 shows that the majority of participants believed the HIV self-test results, were interested in HIV self-testing in the future, and were willing to pay for HIV self-testing. The price that the majority of participants (54%, 53/99) were willing to pay for an HIV self-test was 1000-5000 Ugandan Shillings (UGX) (US $0.3-$1.4); 23% (23/99) of participants reported a willingness to pay 5000-10000 UGX (US $1.4-$2.88) for an HIV self-test, and only 5% (5/99) of participants reported a willingness to pay greater than 10000 UGX (US $2.88) for an HIV self-test.

DISCUSSION

FSWs in urban Uganda, who did not receive pre-test training, had difficulties completing the steps in the oral HIV self-testing process, correctly interpreting self-test results, and understanding the next appropriate steps for HIV treatment and prevention following self-testing. The FSW-interpreted HIV self-test sensitivity and specificity measured in this study is significantly lower than that measured in other studies,[14–23] but consistent with that measured in another Kampala-based FSW population.[25] Despite challenges using and interpreting the HIV self-tests, the majority of FSWs in this study believed the self-test results and were interested and willing to pay for HIV self-tests in the future.

The low measurements of FSW-interpreted HIV self-test sensitivity and specificity and the percentage of participants that correctly identify the next steps for HIV treatment and prevention following HIV self-testing in this study are concerning. False perception of HIV status, attributable to HIV self-testing may result in behaviors or contribute to mental states among FSWs that are counterproductive to health. For example, an incorrectly perceived HIV-positive self-test result may result in depression,[34] while an incorrectly perceived HIV-negative self-test result may delay HIV treatment. Then, even if FSWs correctly learn their HIV status though self-testing, the inability of

For peer review only - http://bmjopen.bmj.com/site/about/guidelines.xhtml

participants in this study to correctly identify the next steps in HIV treatment and prevention following HIV self-testing suggests that HIV self-testing may result in missed opportunities to improve individuals' health and prevent HIV transmission.[35,36]

The low FSW-interpreted HIV self-test sensitivity and specificity measurements in this study may be explained by the lack of pre-test training provided to study participants. Understanding HIV self-test performance in the absence of pre-test training is important because this reflects a potential real-world scenario where FSWs purchase an HIV self-test from a pharmacy or receive one from a friend. Pre-test training interventions are also expensive, especially the intensity of those provided in the other HIV self-testing performance studies,[14–21] and thus difficult to implement at scale in the context of limited health budgets and competing HIV prevention interventions.[24] More research should be conducted to determine the appropriate level of pre-test training necessary for high HIV self-test performance.

Other characteristics of our study population and study design, may have additionally contributed to our low FSW-interpreted HIV self-test sensitivity and specificity measurements. First, our study focused on FSWs, a key population that we hypothesized may have increased difficulties with HIV self-test performance because of often cited lower levels of health literacy [10] and educational attainment [11–13] compared to members of the general population. Lower levels of health literacy and educational attainment, however, is an unlikely explanation for low self-test performance in this study because the majority of participants self-reported the ability to read and write. Second, only 80% of participants in this study removed the buffer cap of the HIV self-test, enabling them to properly HIV self-test. Those who did not properly use the HIV self-test may have had less confidence in their ability interpret self-test results, resulting in increased errors. Third, participants interpreted an HIV self-test result that was not their own and thus interpretations of self-test results were not biased by knowledge of previous HIV test results and past HIV risk encounters.

For peer review only - http://bmjopen.bmj.com/site/about/guidelines.xhtml

There are limitations with this study that are important to note. First, the sample size was small because this study was a sub-study of a larger HIV self-testing randomized controlled trial.[8] Second, the participants in this study were observed using the HIV self-tests instead of testing in isolation, which may have either positively or negatively influenced study results depending on if this made participants more nervous or careful while self-testing or interpreting self-test results.[16] Third, the participants in this study were instructed to not ask research assistants for assistance with the HIV self-testing process or interpreting the HIV self-test results, which might not represent a real life scenario where individuals may seek help with self-testing from friends, partner or others. Fourth, while unlikely, research assistants many have incorrectly drawn the results of random HIV self-tests given to participants for interpretation, potentially resulting in a downward bias of our FSW-interpreted HIV self-test sensitivity and specificity estimates. Fifth, some of the self-tests interpreted by study participants may have been interpreted 40 minutes after use, which is outside of the recommended interpretation period. This many have resulted in a greater number of weak HIV-positive self-test results, which are more difficult to interpret. For this reason, weak HIV-positive self-test results were excluded from our measurement of FSW-interpreted HIV self-test sensitivity. Sixth, participants did not interpret their own HIV self-test results and thus may have been less careful when interpreting a random HIV self-test (especially since many of them likely already knew their HIV status). However, we have previously described the advantages of this study design. Finally, some participants may have been living with HIV and on antiretroviral treatment, which may have biased their perceptions of the next steps following an HIV-positive self-test result and likely resulted in an overestimation of participants reporting that individuals who self-test HIV-positive should start treatment.

The generalizability of the study results may additionally be limited. First, participants in our study used oral HIV self-tests and thus the results from this study do not represent all forms of HIV self-testing, such as blood-based HIV self-tests. Second, participants were instructed to not ask research

For peer review only

assistants for assistance with the HIV self-testing process or interpreting the HIV self-test results, which might not represent a real-life scenario where individuals may seek help with self-testing from friends, sexual partners, or others. Third, the participants in this study were recommended by leaders within the Kampala FSW community and not necessarily representative of the greater Kampala-based FSW population. The FSW peer educators in this study may have higher health literacy and education compared to other FSWs, potentially resulting in greater HIV self-test performance. Our study results may also have limited generalizability among other populations of FSWs, such as those working in rural setting or transit towns, or those who may not identify as FSWs (i.e., barmaids who exchange sex with patrons or young women with 'sugar daddies').[13,26]

CONCLUSIONS

This study demonstrated that FSWs in urban Uganda have difficulty using and interpreting HIV self-test in the absences of pre-test training. The findings from this study suggest that governments considering HIV self-testing need to carefully consider what information FSWs are given before they self-test and what resources will be available to them during or after self-testing to prevent mistakes in the self-testing process, misperceptions about HIV status, and delays in HIV treatment or prevention interventions. The intensity of pre-test training in previous HIV self-test performance studies may be difficult to scale and might not fit within existing national HIV budgets.[24] Simpler HIV self-testing support tools (i.e., instruction guides or demonstration videos), redesign of HIV self-test, or the use of HIV self-testing counselors should be explored and additional research, including randomized controlled trials, should be conducted to determine the appropriate level of pre-test training.

For peer review only - http://bmjopen.bmj.com/site/about/guidelines.xhtml

ACKNOWLEDGMENTS

We would like to acknowledge all the FSWs who took time participating in this study as well as the research assistants who took time collecting this data.

FUNDING STATEMENT

This study was funded by the International Initiative for Impact Evaluation (3ie). KFO was additionally supported by the National Institute of Allergy and Infectious Disease T32-AI007535 (PI: Seage). CEO was supported in part by the National Institute on Drug Abuse T32-DA013911 (PI: Flanigan) and the National Institute of Allergy and Mental Health R25-MH083620 (PI: Nunn). TB was funded by the Alexander von Humboldt Foundation through the Alexander von Humboldt Professorship endowed by the German Federal Ministry of Education and Research. He was also supported by the Wellcome Trust, the European Commission, the Clinton Health Access Initiative and NICHD of NIH R01-HD084233, NIAID of NIH R01-AI124389 and R01-AI112339 and FIC of NIH D43-TW009775. The funders had no role in study design, data collection and analysis, decision to publish, or preparation of the manuscript.

COMPETING INTEREST STATEMENT

The authors of this study declare no conflicts of interest.

INC. CONTRI. STATEMENT

KFO, DKM and CEO designed the study and conceptualized the paper. KFO conducted the analysis and wrote the first draft. KFO, CEO, TN, DKM, GT, LB and AN oversaw the collection and the quality data. CEO and TB supervised the study. KFO, DKM, TN, AN, GT, LB, TB and CEO all edited and revised the manuscript.

For peer review only

DATA SHARING STATEMENT

Data is available from the authors upon request.

For peer review only

For peer review only - http://bmjopen.bmj.com/site/about/guidelines.xhtml

REFERENCES

1 Ayles H, Floyd S, Mulubwa C, *et al.* Increasing knowledge of HIV status among men: a cluster-randomised trial of community-based distribution of oral HIV self-test kits nested in four HPTN 071 communities in Zambia. Paris, France: 2017. http://programme.ias2017.org/Abstract/Abstract/5600

2 Gichangi A, Wambua J, Gohole A, *et al.* Provision of oral HIV self-test kits triples uptake of HIV testing among male partners of antenatal care clients: results of a randomized trial in Kenya. Durban, South Afirca: 2016.

3 Masters SH, Agot K, Obonyo B, *et al.* Promoting Partner Testing and Couples Testing through Secondary Distribution of HIV Self-Tests: A Randomized Clinical Trial. *PLOS Med* 2016;**13**:e1002166. doi:10.1371/journal.pmed.1002166

4 Thirumurthy H, Masters SH, Mavedzenge SN, *et al.* Promoting male partner HIV testing and safer sexual decision making through secondary distribution of self-tests by HIV-negative female sex workers and women receiving antenatal and post-partum care in Kenya: a cohort study. *Lancet HIV* 2016;**3**:e266-274. doi:10.1016/S2352-3018(16)00041-2

5 Johnson CC, Kennedy C, Fonner V, *et al.* Examining the effects of HIV self-testing compared to standard HIV testing services: a systematic review and meta-analysis. *J Int AIDS Soc* 2017;**20**. doi:10.7448/ias.20.1.21594

6 Wang Z, Lau JTF, Ip M, *et al.* A Randomized Controlled Trial Evaluating Efficacy of Promoting a Home-Based HIV Self-Testing with Online Counseling on Increasing HIV Testing Among Men Who Have Sex with Men. *AIDS Behav* Published Online First: 22 August 2017. doi:10.1007/s10461-017-1887-2

For peer review only

For peer review only - http://bmjopen.bmj.com/site/about/guidelines.xhtml

7 Katz D, Golden M, Hughes J, *et al.* HIV self-testing increases HIV testing frequency among high-risk men who have sex with men: a randomized controlled trial. Vancouver, Canada:

8 Ortblad K, Kibuuka Musoke D, Ngabirano T, *et al.* Direct provision versus facility collection of HIV self-tests among female sex workers in Uganda: a cluster-randomized controlled health systems trial. *PLoS Med* 2017;**14**:e1002458.

9 Consolidated Guidelines on HIV Testing Services; 5Cs: Consent, Confidentiality, Counseling, Correct Results and Connection. Geneva, Switzerland: : WHO 2015. http://apps.who.int/iris/bitstream/10665/179870/1/9789241508926_eng.pdf

10 Parker RG, Perez-Brumer A, Garcia J, *et al.* Prevention literacy: community-based advocacy for access and ownership of the HIV prevention toolkit. *J Int AIDS Soc* 2016;**19**. doi:10.7448/IAS.19.1.21092

11 Scorgie F, Chersich MF, Ntaganira I, *et al.* Socio-demographic characteristics and behavioral risk factors of female sex workers in sub-saharan Africa: a systematic review. *AIDS Behav* 2012;**16**:920–33. doi:10.1007/s10461-011-9985-z

12 Ngugi EN, Roth E, Mastin T, *et al.* Female sex workers in Africa: epidemiology overview, data gaps, ways forward. *SAHARA J J Soc Asp HIVAIDS Res Alliance* 2012;**9**:148–53. doi:10.1080/17290376.2012.743825

13 Shannon K, Strathdee SA, Goldenberg SM, *et al.* Global epidemiology of HIV among female sex workers: influence of structural determinants. *Lancet Lond Engl* 2015;**385**:55–71. doi:10.1016/S0140-6736(14)60931-4

For peer review only

14 Choko AT, MacPherson P, Webb EL, *et al.* Uptake, Accuracy, Safety, and Linkage into Care over Two Years of Promoting Annual Self-Testing for HIV in Blantyre, Malawi: A Community-Based Prospective Study. *PLoS Med* 2015;**12**:e1001873. doi:10.1371/journal.pmed.1001873

15 Choko AT, Desmond N, Webb EL, *et al.* The Uptake and Accuracy of Oral Kits for HIV Self-Testing in High HIV Prevalence Setting: A Cross-Sectional Feasibility Study in Blantyre, Malawi. *PLOS Med* 2011;**8**:e1001102. doi:10.1371/journal.pmed.1001102

16 Asiimwe S, Oloya J, Song X, *et al.* Accuracy of un-supervised versus provider-supervised self-administered HIV testing in Uganda: A randomized implementation trial. *AIDS Behav* 2014;**18**:2477–84. doi:10.1007/s10461-014-0765-4

17 Kurth AE, Cleland CM, Chhun N, *et al.* Accuracy and Acceptability of Oral Fluid HIV Self-Testing in a General Adult Population in Kenya. *AIDS Behav* 2016;**20**:870–9. doi:10.1007/s10461-015-1213-9

18 Pai NP, Behlim T, Abrahams L, *et al.* Will an Unsupervised Self-Testing Strategy for HIV Work in Health Care Workers of South Africa? A Cross Sectional Pilot Feasibility Study. *PLOS ONE* 2013;**8**:e79772. doi:10.1371/journal.pone.0079772

19 Dong M, Regina R, Hlongwane S, *et al.* Can laypersons in high prevalence South Africa perform an HIV self-test accurately? *International AIDS Conference* 2014. Melbourne, Australia.

20 Napierala Mavedzenge S, Sibanda E, Mavengere Y, *et al.* Supervised HIV self-testing to inform implementation and scale up of self-testing in Zimbabwe. *J Int AIDS Soc* Published Online First: 1 July 2015. https://www.rti.org/publication/supervised-hiv-self-testing-inform-implementation-and-scale-self-testing-zimbabwe (accessed 20 Mar 2017).

For peer review only

For peer review only - http://bmjopen.bmj.com/site/about/guidelines.xhtml

21 Martínez Pérez G, Steele SJ, Govender I, *et al.* Supervised oral HIV self-testing is accurate in rural KwaZulu-Natal, South Africa. *Trop Med Int Health TM IH* 2016;**21**:759–67. doi:10.1111/tmi.12703

22 Marley G, Kang D, Wilson EC, *et al.* Introducing rapid oral–fluid HIV testing among high risk populations in Shandong, China: feasibility and challenges. *BMC Public Health* 2014;**14**:422. doi:10.1186/1471-2458-14-422

23 Figueroa C, Johnson C, Ford N, *et al.* Reliability of HIV Rapid Diagnostic Tests for Self-Testing Performed by Self-Testers Compared to Healthcare Workers: A Systematic Review and Meta-Analysis. *Lancet HIV* 2018. doi:10.1016/S2352-3018(18)30044-4

24 IHME. Financing Global health 2015: Development assistance steady on the path to new Global Goals. Seattle, WA: Institute for Health Metrics and Evaluation 2016. http://www.healthdata.org/policy-report/financing-global-health-2015-development-assistance-steady-path-new-global-goals (accessed 28 Mar 2017).

25 Ortblad K, Kibuuka Musoke D, Ngabirano T, *et al.* Female sex workers often incorrectly interpret HIV self-test results in Uganda. *JAIDS* 2018 *[In press]*

26 Baral S, Beyrer C, Muessig K, *et al.* Burden of HIV among female sex workers in low-income and middle-income countries: a systematic review and meta-analysis. *Lancet Infect Dis* 2012;**12**:538–49. doi:10.1016/S1473-3099(12)70066-X

27 HIVST.org. Policy & Regulations for HIVST. HIVST.org. http://www.hivst.org/policy-regulations-for-hivst-1/ (accessed 8 Sep 2015).

For peer review only

28 CDC. Crane Survey: Select Results from Recent Surveys, Kampala 2012/3. http://documentslide.com/documents/crane-survey-select-results-from-recent-surveys-kampala-20123-men-who-have.html (accessed 28 Mar 2017).

29 Uganda AIDS Commission. HIV and AIDS Uganda Country Progress Report; 2013. Kampala, Uganda: Uganda AIDS Commission 2014. http://www.uhasselt.be/Documents/UHasselt/onderwijs/internationaal/noord-zuid_2015/HIV_and_AIDS_Uganda_Country_Progress_Report_2013.pdf (accessed 5 Sep 2015).

30 Ugandan Ministry of Health. National HIV Testing Services Policy and Implementation Guidelines Uganda: 4th Edition. Kampala, Uganda: Ministry of Health 2016.

31 Delaney KP, Branson BM, Uniyal A, *et al.* Performance of an oral fluid rapid HIV-1/2 test: experience from four CDC studies. *AIDS Lond Engl* 2006;**20**:1655–60. doi:10.1097/01.aids.0000238412.75324.82

32 Wesolowski LG, MacKellar DA, Facente SN, *et al.* Post-marketing surveillance of OraQuick whole blood and oral fluid rapid HIV testing. *AIDS Lond Engl* 2006;**20**:1661–6. doi:10.1097/01.aids.0000238413.13442.ed

33 Pai NP, Balram B, Shivkumar S, *et al.* Head-to-head comparison of accuracy of a rapid point-of-care HIV test with oral versus whole-blood specimens: a systematic review and meta-analysis. *Lancet Infect Dis* 2012;**12**:373–80. doi:10.1016/S1473-3099(11)70368-1

34 L'akoa RM, Noubiap JJN, Fang Y, *et al.* Prevalence and correlates of depressive symptoms in HIV-positive patients: a cross-sectional study among newly diagnosed patients in Yaoundé, Cameroon. *BMC Psychiatry* 2013;**13**:228. doi:10.1186/1471-244X-13-228

For peer review only

35 Cohen MS, Chen YQ, McCauley M, *et al.* Prevention of HIV-1 Infection with Early Antiretroviral Therapy. *N Engl J Med* 2011;**365**:493–505. doi:10.1056/NEJMoa1105243

36 Donnell D, Baeten JM, Kiarie J, *et al.* Heterosexual HIV-1 transmission after initiation of antiretroviral therapy: a prospective cohort analysis. *The Lancet* 2010;**375**:2092–8. doi:10.1016/S0140-6736(10)60705-2

For peer review only

FIGURE LEGENDS

Figure 1. Participants' interpretations of the HIV self-test results.

Figure 2. Participants' belief in the HIV self-test results, interest in HIV self-testing, and willingness to pay for HIV self-testing.

For peer review only

For peer review only - http://bmjopen.bmj.com/site/about/guidelines.xhtml

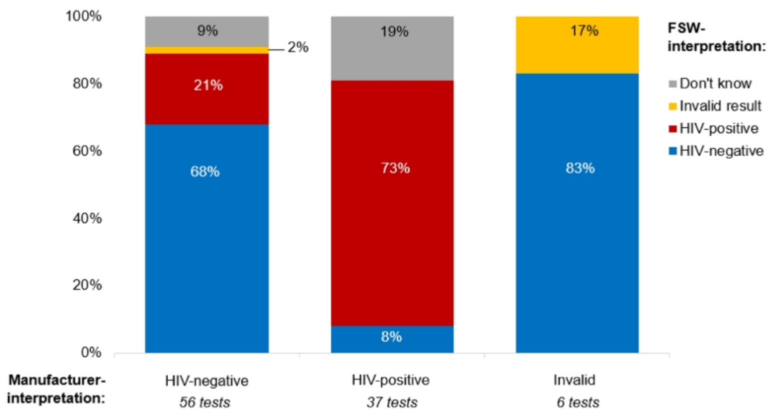

Figure 1. Participants' interpretations of the HIV self-test results.

215x166mm (300 x 300 DPI)

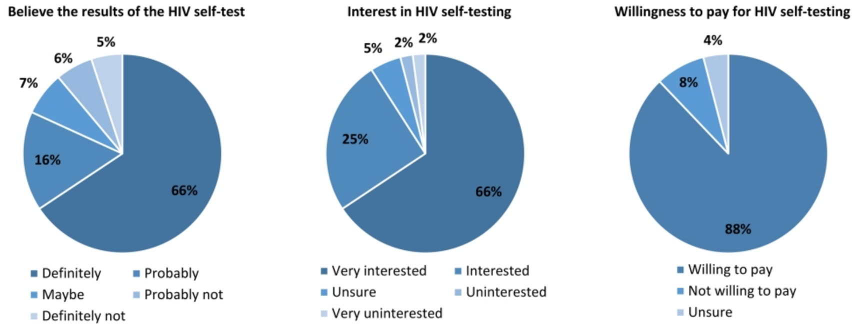

Figure 2. Participants' belief in the HIV self-test results, interest in HIV self-testing, and willingness to pay for HIV self-testing.

215x166mm (300 x 300 DPI)

STROBE Statement—Checklist of items that should be included in reports of ***cross-sectional studies***

	Item No	Recommendation	Page #
**Title and abstract**	1	(*a*) Indicate the study's design with a commonly used term in the title or the abstract	1
		(*b*) Provide in the abstract an informative and balanced summary of what was done and what was found	1
**Introduction**			
Background/rationale	2	Explain the scientific background and rationale for the investigation being reported	4-5
Objectives	3	State specific objectives, including any prespecified hypotheses	5
**Methods**			
Study design	4	Present key elements of study design early in the paper	5-8
Setting	5	Describe the setting, locations, and relevant dates, including periods of recruitment, exposure, follow-up, and data collection	5-9
Participants	6	(*a*) Give the eligibility criteria, and the sources and methods of selection of participants	6
Variables	7	Clearly define all outcomes, exposures, predictors, potential confounders, and effect modifiers. Give diagnostic criteria, if applicable	6-9
Data sources/ measurement	8*	For each variable of interest, give sources of data and details of methods of assessment (measurement). Describe comparability of assessment methods if there is more than one group	6-9
Bias	9	Describe any efforts to address potential sources of bias	8
Study size	10	Explain how the study size was arrived at	6
Quantitative variables	11	Explain how quantitative variables were handled in the analyses. If applicable, describe which groupings were chosen and why	8-9
Statistical methods	12	(*a*) Describe all statistical methods, including those used to control for confounding	8-9
		(*b*) Describe any methods used to examine subgroups and interactions	n/a
		(*c*) Explain how missing data were addressed	n/a
		(*d*) If applicable, describe analytical methods taking account of sampling strategy	n/a
		(*e*) Describe any sensitivity analyses	n/a
**Results**			
Participants	13*	(a) Report numbers of individuals at each stage of study—eg numbers potentially eligible, examined for eligibility, confirmed eligible, included in the study, completing follow-up, and analysed	9
		(b) Give reasons for non-participation at each stage	9
		(c) Consider use of a flow diagram	n/a
Descriptive data	14*	(a) Give characteristics of study participants (eg demographic, clinical, social) and information on exposures and potential confounders	9-10
		(b) Indicate number of participants with missing data for each variable of interest	10
Outcome data	15*	Report numbers of outcome events or summary measures	10-13

For peer review only

Main results	16	(*a*) Give unadjusted estimates and, if applicable, confounder-adjusted estimates and their precision (eg, 95% confidence interval). Make clear which confounders were adjusted for and why they were included	10-13
		(*b*) Report category boundaries when continuous variables were categorized	n/a
		(*c*) If relevant, consider translating estimates of relative risk into absolute risk for a meaningful time period	n/a
Other analyses	17	Report other analyses done—eg analyses of subgroups and interactions, and sensitivity analyses	10-13
**Discussion**			
Key results	18	Summarise key results with reference to study objectives	13-14
Limitations	19	Discuss limitations of the study, taking into account sources of potential bias or imprecision. Discuss both direction and magnitude of any potential bias	15
Interpretation	20	Give a cautious overall interpretation of results considering objectives, limitations, multiplicity of analyses, results from similar studies, and other relevant evidence	15
Generalisability	21	Discuss the generalisability (external validity) of the study results	15-16
**Other information**			
Funding	22	Give the source of funding and the role of the funders for the present study and, if applicable, for the original study on which the present article is based	17

*Give information separately for exposed and unexposed groups.

Note: An Explanation and Elaboration article discusses each checklist item and gives methodological background and published examples of transparent reporting. The STROBE checklist is best used in conjunction with this article (freely available on the Web sites of PLoS Medicine at http://www.plosmedicine.org/, Annals of Internal Medicine at http://www.annals.org/, and Epidemiology at http://www.epidem.com/). Information on the STROBE Initiative is available at www.strobe-statement.org.

For peer review only

BMJ Open

HIV self-test performance among female sex workers in Kampala, Uganda: a cross-sectional study

Journal:	*BMJ Open*
Manuscript ID	bmjopen-2018-022652.R2
Article Type:	Research
Date Submitted by the Author:	03-Jul-2018
Complete List of Authors:	Ortblad, Katrina; Harvard T.H. Chan School of Public Health, Global Health and Population<br>Kibuuka Musoke , Daniel ; International Research Consortium<br>Ngabirano , Thomson ; Uganda Health Marketing Group<br>Nakitende, Aidah ; International Research Consortium<br>Taasi , Geoffrey ; Uganda Ministry of Health<br>Barresi, Leah; Harvard T.H. Chan School of Public Health, Epidemiology<br>Bärnighausen, Till ; Heidelberg University, Institute of Public Health<br>Oldenburg, Catherine; University of California San Francisco, Francis I Proctor Foundation
**Primary Subject Heading**:	HIV/AIDS
Secondary Subject Heading:	Global health, Epidemiology, Public health
Keywords:	HIV self-testing, female sex workers, Uganda, Africa, performance

HIV self-test performance among female sex workers in Kampala, Uganda: a cross-sectional study

Katrina Ortblad[1], Daniel Kibuuka Musoke[2], Thomson Ngabirano[3], Aidah Nakitende[2], Geoffrey Taasi[4], Leah G. Barresi[5], Till Bärnighausen[1,6,7], Catherine E. Oldenburg[8,9,10]

[1]Department of Global Health and Population, Harvard T.H. Chan School of Public Health, Boston, MA, USA
[2]International Research Consortium, Kampala, Uganda
[3]Uganda Health Marketing Group, Kampala, Uganda
[4]Ugandan Ministry of Health, Kampala, Uganda
[5]Department of Epidemiology, Harvard T.H. Chan School of Public Health, Boston, MA, USA
[6]Institute of Public Health, Faculty of Medicine, Heidelberg University, Heidelberg, Germany
[7]Africa Health Research Institute, KwaZulu-Natal, South Africa
[8]Francis I. Proctor Foundation, University of California, San Francisco, San Francisco, CA, USA
[9]Department of Ophthalmology, University of California, San Francisco, San Francisco, CA, USA
[10]Department of Epidemiology and Biostatistics, University of California, San Francisco, San Francisco, CA, USA

Journal: BMJ Open
Word count: 2,784
Abstract Word Count: 225
Tables: 2
Figures: 3
References: 38
Appendix Tables: 1

Corresponding Author:
Katrina F. Ortblad, MPH
Department of Global Health and Population
Harvard T.H. Chan School of Public Health
677 Huntington Ave
Boston, MA 02115
+1-206-265-1856
katrina.ortblad@mail.harvard.edu

Keywords: HIV self-testing; female sex workers; ; performance; Africa; Uganda

For peer review only

ABSTRACT

Objective: To evaluate HIV self-testing performance and results interpretation among female sex workers (FSWs) in Kampala, Uganda who performed unassisted HIV self-testing.

Methods: In October 2016, 104 participants used an oral HIV self-test while under observation by research assistants. Participants were not assisted on HIV self-test use prior to or during testing, and were only given the manufacturer's pictorial and written instructions to guide them. Research assistants recorded if participants completed and/or had difficulties with steps in the HIV self-testing process on a pre-specified checklist. Randomly drawn, used HIV self-tests were interpreted by FSWs. We calculated the concordance between FSWs' interpretations of self-test results with those indicated in the manufacturer's instructions.

Results: Only 33% (34/104) of participants completed all of the key steps in the HIV self-testing process and the majority (86%, 89/104) were observed having difficulties with at least one of these steps. Misinterpretation of HIV self-test results were common among FSWs: 23% (12/56) of FSWs interpreted HIV-negative self-test results as HIV-positive and 8% (3/37) of FSWs interpreted HIV-positive self-test results as HIV-negative. The concordance between FSWs' interpretations of self-test results and that indicated in the instructions was 73% (95%CI 56-86%) for HIV-positive self-tests and 68% (95%CI 54-80%) for HIV-negative self-tests.

Conclusions: FSWs in Kampala, who performed unassisted HIV self-testing, skipped steps in the HIV self-testing process and had difficulties correctly interpreting self-test results. Training on use and interpretation of HIV self-tests may be necessary to prevent errors in the HIV self-testing process and to avoid the negative consequences of false positive and false negative HIV self-test results among FSWs.

For peer review only

For peer review only - http://bmjopen.bmj.com/site/about/guidelines.xhtml

ARTICLE SUMMARY

Strengths and limitations of this study

- This is one of few studies to explore unassisted oral HIV self-test performance among female sex workers, which represents a scenario where female sex workers might obtain HIV self-tests from pharmacies or friends.
- Unlike other studies, female sex workers in this study did not interpret their own HIV self-test result, thus their interpretations were not biased by knowledge of previous HIV test results or HIV risk encounters.
- Study limitations include our small sample size and our limited generalizability of study results to other populations of female sex workers.

For peer review only

INTRODUCTION

HIV self-testing has been shown to increase HIV testing compared to standard of care HIV testing services in diverse populations,[1–8] however, the benefits of HIV self-testing depend on the self-testers ability to correctly follow a sequence of steps, interpret self-test results, and know how to link to appropriate HIV prevention and treatment services. HIV self-testing might be particularly beneficial for female sex workers (FSWs), who are recommended to test for HIV frequently by the World Health Organization (WHO).[9] A recent randomized controlled health systems trial found that HIV self-testing significantly increased recent and repeat HIV testing compared to referral to standard HIV testing services among FSWs in Kampala, Uganda.[8]

Previous HIV self-test performance studies found high sensitivity and specificity of participant-interpreted HIV self-test results.[10–19] In the majority of these studies, however, participants were provided with extensive assistance prior the HIV self-testing.[10–12,14,17] For example, in one study participants were asked to demonstrate their understanding of self-testing with a cotton bud and vial of water,[10] while in another study, fisherfolk received a 10-minute demonstration on how to use the self-test prior to testing.[12] In a real-world setting, this level of assistance would be difficult to implement with declining national HIV budgets and other competing health priorities.[20] In previous HIV self-test performance studies participants interpreted their own HIV self-test result, potentially introducing bias attributable to knowledge of prior HIV test results and HIV risk encounters.[10–18] Understanding how well individuals can interpret HIV self-test results without bias of prior knowledge is important because as HIV self-testing moves HIV testing outside the unregulated environment of the health system,[8] individuals might use an HIV self-test for first time HIV testing. Few studies to date has measured HIV self-test performance among FSWs,[18,21] an important population for HIV prevention interventions.[22,23]

For peer review only - http://bmjopen.bmj.com/site/about/guidelines.xhtml

In this study, we measured unassisted HIV self-test performance (i.e., performance in the absence of any assistance prior to or during HIV self-testing) among FSWs in Kampala, Uganda. Specifically, we explore the feasibility and usability of HIV self-tests among FSWs, including FSWs' ability to complete the sequence of steps necessary for self-testing, interpret HIV self-test results, and identify the next steps for HIV treatment and prevention. Additionally, we explore FSWs' values and preferences for HIV self-test, including acceptability and willingness to pay for HIV self-testing. The results of this study are intended to inform implementation of HIV self-testing among members of a key population during a time when few sub-Saharan African countries have guidelines on HIV self-testing.[24]

METHODS

Ethics

The study was approved by the Institutional Review Boards at the Harvard T.H. Chan School of Public Health and the Mildmay Uganda Research Ethics Committee, it also received special permission from the Ugandan Ministry of Health to introduce a new HIV testing technology.

Setting

Kampala, the capitol city of Uganda, has roughly 13,000 FSWs operating in more than 180 venues. One in every three of these FSWs is estimated to be living with HIV.[25,26] The Ugandan Ministry of Health identifies FSWs as a priority population for HIV prevention interventions [27] and provides them with specialized health services (including clinics and community-based HIV testing) through the Most at Risk Populations Initiative (MARPI).

Participants

For peer review only - http://bmjopen.bmj.com/site/about/guidelines.xhtml
For peer review only

The participants in this study were in training to be peer educators for an HIV self-testing randomized controlled health systems trial among FSWs.[8] We determined the number of peer educators necessary (120) based on our sample and cluster size for the main trial.[8] To recruit FSW peer educators for the trial, we used established FSW peer organizations in Kampala and MARPI team leaders. Peer educators were eligible for participation if they were 18 years or older and were accepted among members of the FSW community. The peer educator training was two days: day one covered trial procedures, information on the role of the peer educator, and HIV and STIs prevention, while day two covered FSW-friendly HIV testing and treatment options in Kampala and oral HIV self-testing (including how to perform the HIV self-test and interpret the test results). This study took place at the end of training day one, prior to any demonstrations of HIV self-testing. Participation in this study was voluntary, and participants were compensated 16,500 UGX (US $4.70) for their time. All participants provided written informed consent in the language in which they were most comfortable. Those who could not write provided consent with a thumbprint.

Usability of HIV self-tests

The participants were silently observed HIV self-testing by research assistants. All participants used the OraQuick ADVANCE Rapid HIV-1/2 Antibody Test (OraSure Technologies, Bethlehem PA), an oral HIV self-test that delivers results in 20 minutes. Testing took place in large, open rooms that were being used for peer educator training. Three to four participants were in a room at a time and spread out (approximately 10 meters apart) so they could not see or hear one another. The participants received no description of the HIV self-testing process or explanation of how to interpret the results prior to testing and were instructed to not ask the research assistant any questions. All participants had access to written and pictorial OraQuick HIV self-test instructions (available in both English and the local language, Luganda), which included information on how to use the self-test as well as information on

For peer review only

For peer review only - http://bmjopen.bmj.com/site/about/guidelines.xhtml

how to interpret the self-test results and link to care. We did not adapt these instructions for use in this study.

Research assistants recorded the steps participants took when using the HIV self-testing on a standardized checklist, based on the OraQuick instructions. The checklist included: (1) removed buffer cap, (2) put buffer in buffer-stand, (3) swapped upper or lower gum, and (4) put test stick in buffer. Research assistants were instructed to indicate if participants had difficulties with any of these HIV self-testing steps. Additionally, research assistants recorded if participants read the HIV self-test instructions and when they read these instructions. Only one research assistant observed each participant as they HIV self-tested. All used HIV self-tests were taken to a back room (where no participants were present) by the study coordinator so that the results could process in private.

Interpretation of HIV self-test results

Once the HIV self-test results had processed, these self-tests (including HIV-negative, HIV-positive, and invalid results) were placed in a large opaque bag by the study coordinator. Research assistants drew a random HIV self-test from the opaque bag for participants to interpret. Participants only interpreted one randomly drawn HIV self-test instead of many so that previous interpretations of HIV self-test results would not bias later interpretations of HIV self-test results. All valid OraQuick results have at least one red band indicating the control. According to the OraQuick HIV self-testing instructions, a single band indicates a nonreactive or negative test, two red bands indicate a reactive or positive test, and no red bands, blurred bands, or no control band indicate an invalid test. Research assistants recorded the results of the HIV self-test they selected by drawing one line, two lines, no lines, or blurred lines on a standardized paper form before handing the HIV self-test to a participant for interpretation. All participants again received the manufacturer's written and pictorial instructions to aid their interpretations.

For peer review only - http://bmjopen.bmj.com/site/about/guidelines.xhtml

None of the participants in this study learned their HIV status as a result study participation and all self-tests were disposed of after study completion. The participants were not given their own self-test to interpret because many already knew their HIV status and this knowledge would bias our measurement of HIV self-test performance. We were additionally concerned about confidentiality of our participants in a group training environment. Participants who wanted to know their status were referred to clinics in Kampala where they could access free HIV testing and counseling services.

Perceptions of HIV self-testing

All participants completed a brief questionnaire after interpreting a random HIV self-test result. This questionnaire asked participants to describe the appropriate next steps in HIV treatment and prevention following hypothetical self-test results. The questionnaire also asked participants to specify their belief in the accuracy of the self-test results, interest in future self-testing, and willingness to pay for HIV self-testing.

Statistical analysis

We calculated the proportion of participants that completed steps in the HIV self-testing process and the proportion of participants that had difficulties with each of these steps. We also used proportions to calculate how participants interpreted HIV-positive, HIV-negative, and invalid HIV self-test results. We derived “correct” interpretations of self-test results using the self-test manufacturer instructions to interpret researcher assistant drawings of the result given to participants. We additionally calculated concordance between participants’ interpretations and the manufacturer’s interpretations of the strong HIV-positive (two distinct bands) and strong HIV-negative (one distinct band) self-test results. We excluded invalid HIV self-test and weak HIV-positive self-test results from our concordance measurements because these testing outcomes are less common and we wanted to focus

For peer review only - http://bmjopen.bmj.com/site/about/guidelines.xhtml

on how well participants could interpret clear results. We did not compare FSW-interpreted HIV self-test results with laboratory blood-based HIV test results, because when interpreted correctly by trained users, oral HIV self-testing has >99% sensitivity and specificity.[28–30] We used Stata 13.1 (Stata Corporation, College Station, TX) to estimate binomial 95% confidence intervals for our concordance measurements. We then calculated the proportion of participants that described their various perceptions of HIV self-testing, including the next steps following HIV self-test results and accuracy of the HIV self-tests.

Participant and public involvement

The research question explored in this study was developed from conversations we had with the Ugandan Ministry of Health and was intended to address one of their concerns related to scaling HIV self-testing. A number of participants were additionally leaders of FSW peer organizations in Kampala and were consulted during the development of the study to gauge their interest in HIV self-testing. These leaders of FSW peer organizations also helped recruitment of study participants and were invited to attend a national dissemination of study results.

RESULTS

In October 2016, 118 FSWs in urban Kampala, Uganda completed day one of the two-day peer educator training. Of these, 104 (88%) agreed to perform an HIV self-test and 99 (84%) interpreted a HIV self-test and completed the questionnaire. Fourteen of the FSWs who participated in day one of the two-day peer educator training refused to perform HIV self-testing and five of the participants who performed HIV self-testing refused to interpret an HIV self-test result.

Table 1 describes socio-demographic characteristics of study participants. The median age of study participants was 33 years (interquartile range: 29 to 27 years). The majority of participants (59%)

For peer review only - http://bmjopen.bmj.com/site/about/guidelines.xhtml

reported completing some level of secondary education (9 to 12 years) and 90% of participants reported the ability to read and write. In Uganda, all public schooling on reading and writing is conducted in English. Almost all participants (83%) had worked as a peer educator at least once before participating in this study, 59% of which had previously worked for Uganda's Most at Risk Population Initiative (also known as MARPI), which we used to help recruit peer educators for this study.

Table 1. Socio-demographic characteristics of study participants. n=104

**Characteristics**	**n (%)**
Age (median, IQR)	33 (29 to 37)
Education	
No formal (0 years)	4 (4%)
Primary/Junior (<9 years)	28 (27%)
Secondary (9-12 years)	61 (59%)
Vocational	5 (5%)
Tertiary	6 (6%)
Can read and write	94 (90%)
Previously worked as a FSW peer educator	86 (83%)
Times works as a peer educator (median, IQR)	2 (1 to 4)

Abbreviations: sample size (n)

Table 2 highlights the percentage of participants who completed and had difficulties with steps in the HIV self-testing process. While the majority of participants completed each of the individual steps (80% removed the buffer cap; 50% put the buffer in the buffer stand; 80% swabbed the upper or lower gum; 74% put the test stick in the buffer), only 33% of participants completed all four of these steps and the majority of participants (86%) were observed by research assistants as having difficulties with at least one of the steps. Of the 89 participants that had difficulties with at least one of the steps, 66% (n=69) were unable to complete at least one the four indicated steps in the self-testing process. The

For peer review only - http://bmjopen.bmj.com/site/about/guidelines.xhtml

majority of participants read the manufacturer's self-test instructions prior to testing (58%) or during the HIV self-testing process (57%), but 49% were observed as having difficulties with the instructions.

Table 2. The percentage of participants that completed and had difficulties with steps in the HIV self-testing process. n=104[1]

Step	Completed n (%)	Had difficulty n (%)
1. Removed buffer cap	80 (80%)	39 (38%)
2. Put buffer in buffer-stand	52 (50%)	72 (69%)
3. Swabbed upper or lower gum	82 (80%)	53 (51%)
4. Put test stick in buffer	77 (74%)	35 (34%)
All steps 1-4[2]	34 (33%)	14 (13%)
Any step 1-4[3]	102 (98%)	89 (86%)
Read instructions prior to testing	61 (58%)	51 (49%)[4]
Referenced instructions during testing	59 (57%)	

Abbreviations: sample size (n)

[1]Denominators vary based on completeness in reporting

[2]Participants were observed completing all steps 1-4 or having difficulties with all steps 1-4.

[3]Participants were observed completing any of the steps 1-4 or having difficulties with any of the steps 1-

[4]Participants were observed having any difficulties with reading the instructions.

Of 99 used HIV self-tests that were randomly distributed to participants for self-interpretation, 93 (94%) tests had valid results and 6 (6%) tests had invalid results (four had no result bands and two had no control band but a single test band). Of the valid tests, 56 (57%) had one control band and were considered HIV-negative, while 37 (37%) had both a control and test band and were considered HIV-positive (five of these had a weak test band and one had a weak control band).

Figure 1 illustrates how participants interpreted the HIV self-test they were distributed, by how the self-test should have been interpreted according to the OraQuick instructions. Of the 56 HIV-

For peer review only - http://bmjopen.bmj.com/site/about/guidelines.xhtml

negative self-test results, participants correctly interpreted 38 (68%) as HIV-negative and incorrectly interpreted 13 (23%) as HIV-positive or invalid. Of the 37 HIV-positive self-test results, participants correctly interpreted 27 (73%) as HIV-positive and incorrectly interpreted three as HIV-negative (8%). Only one of the invalid self-test results was correctly interpreted as such by participants, the rest were incorrectly interpreted by participants as HIV-negative (83%). The concordance between participants' interpretations of self-test results and that indicated in the manufacturer's instructions was 73% (95%CI 56-86%) for HIV-positive self-tests and 68% (95%CI 54-80%) for HIV-negative self-tests.

When participants were asked what the appropriate next steps for HIV treatment and prevention were following a specified HIV self-test result, few provided the responses suggested in the OraQuick instructions (**Table 3**). For example, only 25% (24/99) of participants said they would test again in 3 months following a HIV-negative test result, only 46% (45/99) of participants said they would get a confirmatory test to clinic following a HIV-positive test result, and only 58% (51/99) and 36% (32/99) of participants respectively said they would retake another oral HIV self-test or get confirmatory test at clinic following an invalid test result.

Table 3. Participant perceptions of the next steps following HIV self-test results. N=99

	**HIV self-test result**		
**Next step**	***HIV-negative*** **n (%)**	***HIV-positive*** **n (%)**	***Invalid*** **n (%)**
Test again in 12 months[1]	0	0	0
Test again in 3 months	24 (25%)	3 (3%)	4 (5%)
Retake another oral HIV self-test	8 (8%)	7 (7%)	51 (58%)
Get confirmatory test at clinic	20 (21%)	45 (46%)	32 (36%)
Don't know	2 (2%)	4 (4%)	3 (3%)
Other: Get counseling	-	21 (21%)	1 (1%)
Other: Start treatment	1 (1%)	41 (42%)	2 (2%)

For peer review only - http://bmjopen.bmj.com/site/about/guidelines.xhtml

Other: Use protection	53 (53%)	4 (4%)	3 (3%)
Other: Look after yourself	1 (1%)	-	-
Other: Suicidal thoughts	-	1 (1%)	-
Other: Test again in 1.5 months	-	1 (1%)	-
Other: Consult others	-	-	2 (2%)
Other: Give up on testing	-	-	1 (1%)

Abbreviations: sample size (n); participants did not mention this "other" category (-)

[1]None of the participants responded that they should test for HIV again in 12 months, as indicated with n=0

Figure 2 shows that the majority of participants believed the HIV self-test results, were interested in HIV self-testing in the future, and were willing to pay for HIV self-testing. The price that the majority of participants (54%, 53/99) were willing to pay for an HIV self-test was 1000-5000 Ugandan Shillings (UGX) (US $0.3-$1.4); 23% (23/99) of participants reported a willingness to pay 5000-10000 UGX (US $1.4-$2.88) for an HIV self-test, and only 5% (5/99) of participants reported a willingness to pay greater than 10000 UGX (US $2.88) for an HIV self-test.

DISCUSSION

FSWs in urban Uganda, who performed unassisted HIV self-testing, had difficulties completing the steps in the oral HIV self-testing process, correctly interpreting self-test results, and understanding the next appropriate steps for HIV treatment and prevention following self-testing. The concordance between FSWs' interpretations and the manufacturer's interpretations of strong HIV-positive and strong HIV-negative self-test results in this study is significantly lower than HIV self-test sensitivity and specificity measurements from other studies,[10–19] but consistent with that measured in another Kampala-based FSW population.[21] Despite challenges using and interpreting the HIV self-tests, the

For peer review only - http://bmjopen.bmj.com/site/about/guidelines.xhtml

majority of FSWs in this study believed the self-test results and were interested and willing to pay for HIV self-tests in the future.

The low concordance between FSWs' interpretations and the manufacturer's interpretations of strong HIV-positive and strong HIV-negative self-test results and the low percentage of participants that correctly identify the next steps for HIV treatment and prevention following HIV self-testing in this study are concerning. False perception of HIV status, attributable to HIV self-testing may result in behaviors or contribute to mental states among FSWs that are counterproductive to health. For example, an incorrectly perceived HIV-positive self-test result may result in depression,[31] while an incorrectly perceived HIV-negative self-test result may delay HIV treatment. Then, even if FSWs correctly learn their HIV status though self-testing, the inability of participants in this study to correctly identify the next steps in HIV treatment and prevention following HIV self-testing suggests that HIV self-testing may result in missed opportunities to improve individuals' health and prevent HIV transmission.[32,33]

The low concordance between FSWs' interpretations and the manufacturer's interpretations of self-test results in this study may be explained by the lack of HIV self-testing assistance provided to study participants. Understanding unassisted HIV self-test performance is important because this reflects a scenario where FSWs may purchase an HIV self-test from a pharmacy or receive one from a friend. Assisted HIV self-testing is also expensive, especially the intensity of those provided in the other HIV self-testing performance studies,[10–17] and thus difficult to implement at scale in the context of limited health budgets and competing HIV prevention interventions.[20] More research should be conducted to determine the appropriate level of assistance, either prior to or during the self-testing process, to achieve high HIV self-test performance.

This study had a number of strengths and limitations. An important strength of this study is that participants interpreted an HIV self-test result that was not their own and thus interpretations of self-

For peer review only - http://bmjopen.bmj.com/site/about/guidelines.xhtml

test results were not biased by knowledge of previous HIV test results and past HIV risk encounters. Another strength was that participants performed unassisted HIV self-testing, which enables us to understand HIV self-testing performance among FSWs in environments where assistance might be unavailable. Limitations of this study, however, included sample size, which was small because this study was a sub-study of a larger HIV self-testing randomized controlled trial.[8] In this study participants were also observed using the HIV self-tests instead of testing in isolation, which may have either positively or negatively influenced study results depending on if this made participants more nervous or careful while self-testing or interpreting self-test results.[12] While unlikely, research assistants many have incorrectly drawn the results of random HIV self-tests given to participants for interpretation, potentially resulting in a downward bias of our concordance measurements. Some of the self-tests interpreted by study participants may have been interpreted 40 minutes after use, which is outside of the recommended interpretation period. This many have resulted in a greater number of weak HIV-positive self-test results, which are more difficult to interpret. For this reason, weak HIV-positive self-test results were excluded from our concordance measurements. Finally, some participants may have been living with HIV and on antiretroviral treatment, which may have biased their perceptions of the next steps following an HIV-positive self-test result and likely resulted in an overestimation of participants reporting that individuals who self-test HIV-positive should start treatment.

The generalizability of the study results may additionally be limited. First, participants in our study used oral HIV self-tests and thus the results from this study do not represent all forms of HIV self-testing, such as blood-based HIV self-tests. Second, participants were instructed to not ask research assistants for assistance with the HIV self-testing process or interpreting the HIV self-test results, which might not represent a scenario where individuals may seek help with self-testing from friends, sexual partners, or others. Third, the participants in this study were recommended by leaders within the Kampala FSW community and not necessarily representative of the greater Kampala-based FSW

For peer review only

For peer review only - http://bmjopen.bmj.com/site/about/guidelines.xhtml

population. The FSW peer educators in this study may have higher health literacy and education compared to other FSWs, potentially resulting in greater HIV self-test performance. Our study results may also have limited generalizability among other populations of FSWs, such as those working in rural setting or transit towns, or those who may not identify as FSWs (i.e., barmaids who exchange sex with patrons or young women with 'sugar daddies').[22,23]

CONCLUSIONS

This study demonstrated that FSWs in urban Uganda have difficulties with the HIV self-testing process and have difficulties interpreting HIV self-test results when unassisted. The findings from this study suggest that governments considering HIV self-testing need to carefully consider what information FSWs are given before they self-test and what resources will be available to them during or after self-testing to prevent mistakes in the self-testing process, misperceptions about HIV status, and delays in HIV treatment or prevention interventions. The intensity of HIV self-testing assistance in previous performance studies may be difficult to scale and might not fit within existing national HIV budgets.[20] Simpler HIV self-testing support tools (i.e., instructions or demonstration videos), redesign of HIV self-test, or the use of HIV self-testing counselors should be explored and additional research should be conducted to determine the appropriate level of HIV self-testing assistance.

For peer review only

For peer review only - http://bmjopen.bmj.com/site/about/guidelines.xhtml

ACKNOWLEDGMENTS

We would like to acknowledge all the FSWs who took time participating in this study as well as the research assistants who took time collecting this data.

FUNDING STATEMENT

This study was funded by the International Initiative for Impact Evaluation (3ie). KFO was additionally supported by the National Institute of Allergy and Infectious Disease T32-AI007535 (PI: Seage). CEO was supported in part by the National Institute on Drug Abuse T32-DA013911 (PI: Flanigan) and the National Institute of Allergy and Mental Health R25-MH083620 (PI: Nunn). TB was funded by the Alexander von Humboldt Foundation through the Alexander von Humboldt Professorship endowed by the German Federal Ministry of Education and Research. He was also supported by the Wellcome Trust, the European Commission, the Clinton Health Access Initiative and NICHD of NIH R01-HD084233, NIAID of NIH R01-AI124389 and R01-AI112339 and FIC of NIH D43-TW009775. The funders had no role in study design, data collection and analysis, decision to publish, or preparation of the manuscript.

COMPETING INTEREST STATEMENT

The authors of this study declare no conflicts of interest.

INC. CONTRI. STATEMENT

KFO, DKM and CEO designed the study and conceptualized the paper. KFO conducted the analysis and wrote the first draft. KFO, CEO, TN, DKM, GT, LB and AN oversaw the collection and the quality data. CEO and TB supervised the study. KFO, DKM, TN, AN, GT, LB, TB and CEO all edited and revised the manuscript.

For peer review only

DATA SHARING STATEMENT

Data is available from the authors upon request.

For peer review only

For peer review only - http://bmjopen.bmj.com/site/about/guidelines.xhtml

REFERENCES

1 Ayles H, Floyd S, Mulubwa C, *et al.* Increasing knowledge of HIV status among men: a cluster-randomised trial of community-based distribution of oral HIV self-test kits nested in four HPTN 071 communities in Zambia. *9th IAS Conference on HIV Science*; July 23-26, 2017; Paris, France.

2 Gichangi A, Wambua J, Gohole A, *et al.* Provision of oral HIV self-test kits triples uptake of HIV testing among male partners of antenatal care clients: results of a randomized trial in Kenya. Durban, South Afirca: 2016.

3 Masters SH, Agot K, Obonyo B, *et al.* Promoting Partner Testing and Couples Testing through Secondary Distribution of HIV Self-Tests: A Randomized Clinical Trial. *PLOS Med* 2016;**13**:e1002166. doi:10.1371/journal.pmed.1002166

4 Thirumurthy H, Masters SH, Mavedzenge SN, *et al.* Promoting male partner HIV testing and safer sexual decision making through secondary distribution of self-tests by HIV-negative female sex workers and women receiving antenatal and post-partum care in Kenya: a cohort study. *Lancet HIV* 2016;**3**:e266-274. doi:10.1016/S2352-3018(16)00041-2

5 Johnson CC, Kennedy C, Fonner V, *et al.* Examining the effects of HIV self-testing compared to standard HIV testing services: a systematic review and meta-analysis. *J Int AIDS Soc* 2017;**20**. doi:10.7448/ias.20.1.21594

6 Wang Z, Lau JTF, Ip M, *et al.* A Randomized Controlled Trial Evaluating Efficacy of Promoting a Home-Based HIV Self-Testing with Online Counseling on Increasing HIV Testing Among Men Who Have Sex with Men. *AIDS Behav* Published Online First: 22 August 2017. doi:10.1007/s10461-017-1887-2

For peer review only

7 Katz D, Golden M, Hughes J, *et al.* HIV self-testing increases HIV testing frequency among high-risk men who have sex with men: a randomized controlled trial. *8th Conference on HIV Pathogenesis, Treatment and Prevention*. July 19-22, 2015. Vancouver, Canada.

8 Ortblad K, Kibuuka Musoke D, Ngabirano T, *et al.* Direct provision versus facility collection of HIV self-tests among female sex workers in Uganda: a cluster-randomized controlled health systems trial. *PLoS Med* 2017;**14**:e1002458.

9 WHO. Consolidated Guidelines on HIV Testing Services; 5Cs: Consent, Confidentiality, Counseling, Correct Results and Connection. Geneva, Switzerland: WHO 2015. http://apps.who.int/iris/bitstream/10665/179870/1/9789241508926_eng.pdf

10 Choko AT, MacPherson P, Webb EL, *et al.* Uptake, Accuracy, Safety, and Linkage into Care over Two Years of Promoting Annual Self-Testing for HIV in Blantyre, Malawi: A Community-Based Prospective Study. *PLoS Med* 2015;**12**:e1001873. doi:10.1371/journal.pmed.1001873

11 Choko AT, Desmond N, Webb EL, *et al.* The Uptake and Accuracy of Oral Kits for HIV Self-Testing in High HIV Prevalence Setting: A Cross-Sectional Feasibility Study in Blantyre, Malawi. *PLOS Med* 2011;**8**:e1001102. doi:10.1371/journal.pmed.1001102

12 Asiimwe S, Oloya J, Song X, *et al.* Accuracy of un-supervised versus provider-supervised self-administered HIV testing in Uganda: A randomized implementation trial. *AIDS Behav* 2014;**18**:2477–84. doi:10.1007/s10461-014-0765-4

13 Kurth AE, Cleland CM, Chhun N, *et al.* Accuracy and Acceptability of Oral Fluid HIV Self-Testing in a General Adult Population in Kenya. *AIDS Behav* 2016;**20**:870–9. doi:10.1007/s10461-015-1213-9

For peer review only

14 Pai NP, Behlim T, Abrahams L, *et al.* Will an Unsupervised Self-Testing Strategy for HIV Work in Health Care Workers of South Africa? A Cross Sectional Pilot Feasibility Study. *PLOS ONE* 2013;**8**:e79772. doi:10.1371/journal.pone.0079772

15 Dong M, Regina R, Hlongwane S, *et al.* Can laypersons in highprevalence South Africa perform an HIV sefl-test accurately? Melbourne, Australia: 2014.

16 Napierala Mavedzenge S, Sibanda E, Mavengere Y, *et al.* Supervised HIV self-testing to inform implementation and scale up of self-testing in Zimbabwe. *J Int AIDS Soc* Published Online First: 1 July 2015.https://www.rti.org/publication/supervised-hiv-self-testing-inform-implementation-and-scale-self-testing-zimbabwe (accessed 20 Mar 2017).

17 Martínez Pérez G, Steele SJ, Govender I, *et al.* Supervised oral HIV self-testing is accurate in rural KwaZulu-Natal, South Africa. *Trop Med Int Health TM IH* 2016;**21**:759–67. doi:10.1111/tmi.12703

18 Marley G, Kang D, Wilson EC, *et al.* Introducing rapid oral–fluid HIV testing among high risk populations in Shandong, China: feasibility and challenges. *BMC Public Health* 2014;**14**:422. doi:10.1186/1471-2458-14-422

19 Figueroa C, Johnson C, Ford N, *et al.* Reliability of HIV Rapid Diagnostic Tests for Self-Testing Performed by Self-Testers Compared to Healthcare Workers: A Systematic Review and Meta-Analysis. *Rev* 2017.

20 Financing Global health 2015: Development assistance steady on the path to new Global Goals. Seattle, WA: : Insitution for Health Metrics and Evalution 2016. http://www.healthdata.org/policy-report/financing-global-health-2015-development-assistance-steady-path-new-global-goals (accessed 28 Mar 2017).

For peer review only

21 Ortblad K, Kibuuka Musoke D, Ngabirano T, *et al.* Female sex workers often incorrectly interpret HIV self-test results in Uganda. *JAIDS*

22 Baral S, Beyrer C, Muessig K, *et al.* Burden of HIV among female sex workers in low-income and middle-income countries: a systematic review and meta-analysis. *Lancet Infect Dis* 2012;**12**:538–49. doi:10.1016/S1473-3099(12)70066-X

23 Shannon K, Strathdee SA, Goldenberg SM, *et al.* Global epidemiology of HIV among female sex workers: influence of structural determinants. *Lancet Lond Engl* 2015;**385**:55–71. doi:10.1016/S0140-6736(14)60931-4

24 HIVST.org. Policy & Regulations for HIVST. HIVST.org. http://www.hivst.org/policy-regulations-for-hivst-1/ (accessed 8 Sep 2015).

25 CDC. Crane Survey: Select Results from Recent Surveys, Kampala 2012/3. http://documentslide.com/documents/crane-survey-select-results-from-recent-surveys-kampala-20123-men-who-have.html (accessed 28 Mar 2017).

26 Ugandan Ministry of Health. HIV and AIDS Uganda Country Progress Report; 2013. Kampala, Uganda: Uganda AIDS Commission 2014. http://www.uhasselt.be/Documents/UHasselt/onderwijs/internationaal/noord-zuid_2015/HIV_and_AIDS_Uganda_Country_Progress_Report_2013.pdf (accessed 5 Sep 2015).

27 Ugandan Ministry of Health. National HIV Testing Services Policy and Implementation Guidelines Uganda: 4th Edition. Kampala, Uganda: Ministry of Health 2016.

28 Delaney KP, Branson BM, Uniyal A, *et al.* Performance of an oral fluid rapid HIV-1/2 test: experience from four CDC studies. *AIDS Lond Engl* 2006;**20**:1655–60. doi:10.1097/01.aids.0000238412.75324.82

For peer review only

29 Wesolowski LG, MacKellar DA, Facente SN, *et al.* Post-marketing surveillance of OraQuick whole blood and oral fluid rapid HIV testing. *AIDS Lond Engl* 2006;**20**:1661–6. doi:10.1097/01.aids.0000238413.13442.ed

30 Pai NP, Balram B, Shivkumar S, *et al.* Head-to-head comparison of accuracy of a rapid point-of-care HIV test with oral versus whole-blood specimens: a systematic review and meta-analysis. *Lancet Infect Dis* 2012;**12**:373–80. doi:10.1016/S1473-3099(11)70368-1

31 L'akoa RM, Noubiap JJN, Fang Y, *et al.* Prevalence and correlates of depressive symptoms in HIV-positive patients: a cross-sectional study among newly diagnosed patients in Yaoundé, Cameroon. *BMC Psychiatry* 2013;**13**:228. doi:10.1186/1471-244X-13-228

32 Cohen MS, Chen YQ, McCauley M, *et al.* Prevention of HIV-1 Infection with Early Antiretroviral Therapy. *N Engl J Med* 2011;**365**:493–505. doi:10.1056/NEJMoa1105243

33 Donnell D, Baeten JM, Kiarie J, *et al.* Heterosexual HIV-1 transmission after initiation of antiretroviral therapy: a prospective cohort analysis. *The Lancet* 2010;**375**:2092–8. doi:10.1016/S0140-6736(10)60705-2

For peer review only

For peer review only - http://bmjopen.bmj.com/site/about/guidelines.xhtml

FIGURE LEGENDS

Figure 1. Participants' interpretations of the HIV self-test results.

Figure 2. Participants' belief in the HIV self-test results, interest in HIV self-testing, and willingness to pay for HIV self-testing.

For peer review only

For peer review only - http://bmjopen.bmj.com/site/about/guidelines.xhtml

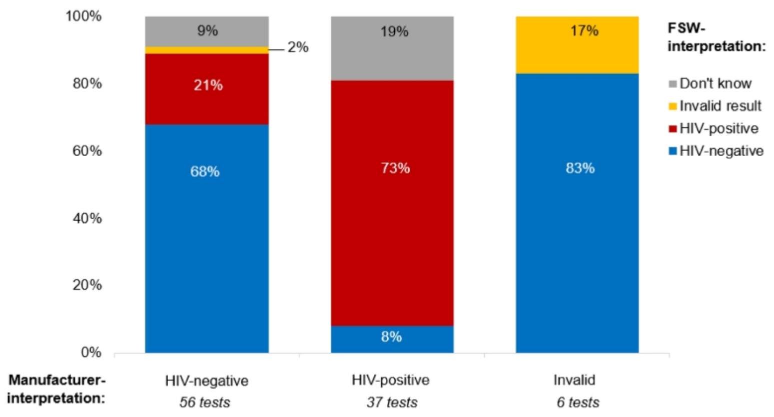

Figure 1. Participants' interpretations of the HIV self-test results.

215x166mm (300 x 300 DPI)

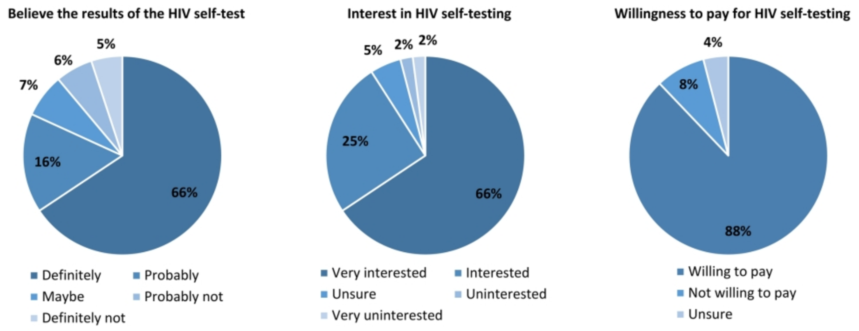

Figure 2. Participants' belief in the HIV self-test results, interest in HIV self-testing, and willingness to pay for HIV self-testing.

215x166mm (300 x 300 DPI)

STROBE Statement—Checklist of items that should be included in reports of ***cross-sectional studies***

	Item No	Recommendation	Page #
**Title and abstract**	1	(*a*) Indicate the study's design with a commonly used term in the title or the abstract	1
		(*b*) Provide in the abstract an informative and balanced summary of what was done and what was found	1
**Introduction**			
Background/rationale	2	Explain the scientific background and rationale for the investigation being reported	4-5
Objectives	3	State specific objectives, including any prespecified hypotheses	5
**Methods**			
Study design	4	Present key elements of study design early in the paper	5-8
Setting	5	Describe the setting, locations, and relevant dates, including periods of recruitment, exposure, follow-up, and data collection	5-9
Participants	6	(*a*) Give the eligibility criteria, and the sources and methods of selection of participants	6
Variables	7	Clearly define all outcomes, exposures, predictors, potential confounders, and effect modifiers. Give diagnostic criteria, if applicable	6-9
Data sources/ measurement	8*	For each variable of interest, give sources of data and details of methods of assessment (measurement). Describe comparability of assessment methods if there is more than one group	6-9
Bias	9	Describe any efforts to address potential sources of bias	8
Study size	10	Explain how the study size was arrived at	6
Quantitative variables	11	Explain how quantitative variables were handled in the analyses. If applicable, describe which groupings were chosen and why	8-9
Statistical methods	12	(*a*) Describe all statistical methods, including those used to control for confounding	8-9
		(*b*) Describe any methods used to examine subgroups and interactions	n/a
		(*c*) Explain how missing data were addressed	n/a
		(*d*) If applicable, describe analytical methods taking account of sampling strategy	n/a
		(*e*) Describe any sensitivity analyses	n/a
**Results**			
Participants	13*	(a) Report numbers of individuals at each stage of study—eg numbers potentially eligible, examined for eligibility, confirmed eligible, included in the study, completing follow-up, and analysed	9
		(b) Give reasons for non-participation at each stage	9
		(c) Consider use of a flow diagram	n/a
Descriptive data	14*	(a) Give characteristics of study participants (eg demographic, clinical, social) and information on exposures and potential confounders	9-10
		(b) Indicate number of participants with missing data for each variable of interest	10
Outcome data	15*	Report numbers of outcome events or summary measures	10-13

For peer review only

Main results	16	(*a*) Give unadjusted estimates and, if applicable, confounder-adjusted estimates and their precision (eg, 95% confidence interval). Make clear which confounders were adjusted for and why they were included	10-13
		(*b*) Report category boundaries when continuous variables were categorized	n/a
		(*c*) If relevant, consider translating estimates of relative risk into absolute risk for a meaningful time period	n/a
Other analyses	17	Report other analyses done—eg analyses of subgroups and interactions, and sensitivity analyses	10-13
**Discussion**			
Key results	18	Summarise key results with reference to study objectives	13-14
Limitations	19	Discuss limitations of the study, taking into account sources of potential bias or imprecision. Discuss both direction and magnitude of any potential bias	15
Interpretation	20	Give a cautious overall interpretation of results considering objectives, limitations, multiplicity of analyses, results from similar studies, and other relevant evidence	15
Generalisability	21	Discuss the generalisability (external validity) of the study results	15-16
**Other information**			
Funding	22	Give the source of funding and the role of the funders for the present study and, if applicable, for the original study on which the present article is based	17

*Give information separately for exposed and unexposed groups.

Note: An Explanation and Elaboration article discusses each checklist item and gives methodological background and published examples of transparent reporting. The STROBE checklist is best used in conjunction with this article (freely available on the Web sites of PLoS Medicine at http://www.plosmedicine.org/, Annals of Internal Medicine at http://www.annals.org/, and Epidemiology at http://www.epidem.com/). Information on the STROBE Initiative is available at www.strobe-statement.org.

For peer review only
